# Serial platelet count as a dynamic prediction marker of hospital mortality among septic patients

**DOI:** 10.1093/burnst/tkae016

**Published:** 2024-06-15

**Authors:** Qian Ye, Xuan Wang, Xiaoshuang Xu, Jiajin Chen, David C Christiani, Feng Chen, Ruyang Zhang, Yongyue Wei

**Affiliations:** Department of Biostatistics, School of Public Health, Nanjing Medical University, 101 Longmian Avenue, Nanjing, Jiangsu 211166, China; Department of Biostatistics, School of Public Health, Nanjing Medical University, 101 Longmian Avenue, Nanjing, Jiangsu 211166, China; Department of Biostatistics, School of Public Health, Nanjing Medical University, 101 Longmian Avenue, Nanjing, Jiangsu 211166, China; Department of Biostatistics, School of Public Health, Nanjing Medical University, 101 Longmian Avenue, Nanjing, Jiangsu 211166, China; Department of Environmental Health, Harvard T.H. Chan School of Public Health, Harvard University, 655 Huntington Avenue, Boston, MA 02115, USA; Pulmonary and Critical Care Division, Massachusetts General Hospital, Department of Medicine, Harvard Medical School, 55 Fruit Street, Boston, MA 02114, USA; Department of Biostatistics, School of Public Health, Nanjing Medical University, 101 Longmian Avenue, Nanjing, Jiangsu 211166, China; Jiangsu Key Lab of Cancer Biomarkers, Prevention and Treatment, Jiangsu Collaborative Innovation Center for Cancer Personalized Medicine, Nanjing Medical University, 101 Longmian Avenue, Nanjing, Jiangsu 211166, China; China International Cooperation Center of Environment and Human Health, Nanjing Medical University, 101 Longmian Avenue, Nanjing, Jiangsu 211166, China; Department of Biostatistics, School of Public Health, Nanjing Medical University, 101 Longmian Avenue, Nanjing, Jiangsu 211166, China; Department of Biostatistics, School of Public Health, Nanjing Medical University, 101 Longmian Avenue, Nanjing, Jiangsu 211166, China; Center for Public Health and Epidemic Preparedness & Response, Peking University, Key Laboratory of Epidemiology of Major Diseases (Peking University), Ministry of Education, 38 Xueyuan Road, Haidian District, Beijing 100191, China

**Keywords:** Sepsis, Platelet count trajectory, Joint latent class model, Dynamic prediction, Intensive care medicine

## Abstract

**Background:**

Platelets play a critical role in hemostasis and inflammatory diseases. Low platelet count and activity have been reported to be associated with unfavorable prognosis. This study aims to explore the relationship between dynamics in platelet count and in-hospital morality among septic patients and to provide real-time updates on mortality risk to achieve dynamic prediction.

**Methods:**

We conducted a multi-cohort, retrospective, observational study that encompasses data on septic patients in the eICU Collaborative Research Database (eICU-CRD) and the Medical Information Mart for Intensive Care IV (MIMIC-IV) database. The joint latent class model (JLCM) was utilized to identify heterogenous platelet count trajectories over time among septic patients. We assessed the association between different trajectory patterns and 28-day in-hospital mortality using a piecewise Cox hazard model within each trajectory. We evaluated the performance of our dynamic prediction model through area under the receiver operating characteristic curve, concordance index (C-index), accuracy, sensitivity, and specificity calculated at predefined time points.

**Results:**

Four subgroups of platelet count trajectories were identified that correspond to distinct in-hospital mortality risk. Including platelet count did not significantly enhance prediction accuracy at early stages (day 1 C-index_Dynamic_  *vs* C-index_Weibull_: 0.713 *vs* 0.714). However, our model showed superior performance to the static survival model over time (day 14 C-index_Dynamic_  *vs* C-index_Weibull_: 0.644 *vs* 0.617).

**Conclusions:**

For septic patients in an intensive care unit, the rapid decline in platelet counts is a critical prognostic factor, and serial platelet measures are associated with prognosis.

Highlights

- Intensive care unit septic patients exhibit significant heterogeneity in platelet count trajectories, with distinct subgroups showing significant stratification in mortality risk.
- For intensive care unit patients with septic shock, rapid decline in platelet count in the early stages and persistent thrombocytopenia are important prognostic factors.
- Real-time updates of risk based on repeated platelet count measurements may enhance further the predictive capability of the model.

## Background

Sepsis is a life-threatening condition caused by dysregulated host response to infection, leading to systemic inflammation, tissue damage and organ failure. It is also one of the leading causes of intensive care unit (ICU) admission and mortality [1]. Platelets play a critical role in clotting, inflammation and immune responses, all of which are linked to the occurrence, development and prognosis of sepsis [2–4].

Previous studies showed that decreasing platelet count during ICU stay was related to higher risk of mortality in septic patients [5–7],and the association between dynamic trajectory patterns of platelet count after ICU admission and mortality has been studied extensively [8–11]. Septic patients often exhibit abnormal activation and over-consumption of platelets, which subsequently lead to thrombocytopenia, thereby increasing bleeding risk [12]. Results from multiple studies have described the prognostic value of early changes in platelet count in septic patients [11, 13]. However, the relationship between trajectories of repeated measures of platelet count collected from daily clinical practice and the prognosis of septic patients is not yet clear.

The aim of this study is to identify heterogenous platelet count trajectories by leveraging the public electronic health record using the joint latent class model (JLCM), which integrates a latent class mixed model with a survival model, and to explore the relationship between platelet dynamics and an unfavorable prognosis.

## Methods

### Study design and populations

This study is a multi-cohort, retrospective, observational study based on data from the eICU Collaborative Research Database (eICU-CRD) v2.0 and the Medical Information Mart for Intensive Care IV (MIMIC-IV) v2.0 database available at PhysioNet (certification number: 49953233). We extracted patients diagnosed with sepsis 24 hours prior to or 48 hours after ICU admission, consistent with a previous study [11], patients were excluded if they met any of the following criteria: (1) repeated ICU admissions; (2) age <18 years; (3) length of ICU stay <24 h; (4) missing baseline platelet count; and (5) platelet count measurements <2 times within 28 days of ICU admission. Ultimately, a total of 11,016 ICU septic patients from eICU-CRD and 7,796 from MIMIC-IV were included in our analysis. Patients from eICU-CRD were included as the discovery set while septic patients from MIMIC-IV were included in the validation set.

### Laboratory measurements and clinical characteristics

Longitudinal platelet counts measured continuously over a 28 day period after ICU admission were collected from both databases. We extracted variables including demographic information, laboratory tests, vital signs, comorbidities and interventions during ICU stay.

### Outcomes

The outcome is survival status and corresponding survival time within 28days of hospital stay. Survival time was defined as the time from ICU admission to either death or loss to follow-up at the end of the study, whichever occurred first. Patients who were discharged from the hospital within 28 days or who remained alive after 28 days were considered censored.

### Statistical analysis

#### Step 1: identification of subpopulations

JLCM was used for identifying subpopulations with heterogenous platelet trajectories in eICU-CRD. For the longitudinal model, a latent class mixed model was fitted. We used a parametric survival model with the baseline hazard function following a class-specific Weibull distribution. Baseline platelets, acute physiology score III (APS III) and the Charlson comorbidity index (CCI) were included as covariates. The optimal number of classes was determined by Akaike information criterion (AIC), Bayesian information criteria (BIC), sample-adjusted BIC (SABIC) and entropy [14]. The R package *lcmm* (version 2.0.2) was used for modeling.

#### Step 2: trajectories visualization

We compared the laboratory measurements and clinical characteristics in four classes. Corresponding trajectory patterns along with Kaplan-Meier (KM) curves were given. Furthermore, we estimated the time-dependent hazard ratio (HR) using a piecewise Cox regression model; the cut point for generating time interval was determined by searching for the highest log-likelihood value [15, 16].

#### Step 3: model evaluation and comparison

JLCM can estimate survival probability dynamically at given days of follow-up (landmark time) till any time point after that, so the model could be used for dynamic prediction. We compared our model developed from step 1 with a Weibull survival model using the same set of covariates, including APS III, CCI and baseline platelet count. Each day between the first day of ICU admission till day 14 was defined as a landmark time point. Predicted probability from landmark time till day 28 among patients still alive at the landmark time were derived from both models. Patients who experienced the outcome before the landmark time were excluded from the analysis of that particular landmark time [17].

We used time-dependent area under receiver operating characteristic curve (AUC), concordance index (C-index), accuracy, sensitivity and specificity at different landmark times to evaluate the performance of the dynamic prediction model. The 95% confidence intervals (CI) for AUCs and C-indices were obtained using 2000 times bootstrap, and the comparison was performed using permutation tests by shuffling 2000 times at each landmark time.

#### Step 4: individual prediction

For individual dynamic prediction, the model was applied in two patients. Also, we compared the Weibull survival model with our model under the setting of individual prediction. For continuous variables, the Kolmogorov-Smirnov test was used to test normality. Normally distributed continuous variables were represented with mean and standard deviation (SD). Group comparisons were conducted using t-test or analysis of variance. For data that followed a non-normal distribution, median and interquartile range were used; Mann-Whitney test and the Kruskal-Wallis test were applied for group comparisons according to the number of groups. A two-tailed *P* value < 0.05 was considered statistically significant. Data was extracted using PostgreSQL 14 and all statistical analyses were performed using R (version 4.1.2). Detailed descriptions of statistical methods are provided in the supplementary methods file (see online supplementary material).

## Results

### Baseline characteristics of study populations

**Figure 1:**
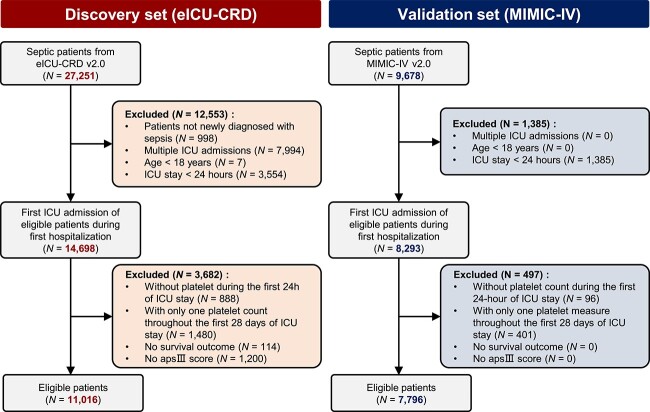
Flowchart illustrating the process of patient selection. *eICU-CRD* eICU Collaborative Research Database, *MIMIC-IV* Medical Information Mart for Intensive Care IV

We analyzed a total of 11,016 septic patients from the eICU-CRD, 19.4% (2,138 patients) of the patients died before hospital discharge. For MIMIC-IV, 7796 patients passed the exclusion criteria and 19.3% (1,507 patients) died during hospital stay. Figure 1 illustrates the patient exclusion process, while Table 1 summarizes the baseline characteristics of patients in two datasets; additional clinical characteristics are provided in Table S1 (see online supplementary material).

**Table 1:** Clinical baseline characteristics of patients in eICU-CRD and MIMIC-IV database

	**eICU-CRD v2.0** **(N = 11 016)**	**MIMIC-IV v2.0** **(N = 7796)**
**Basic characteristics**		
Age (years), median [IQR]	68.00 [57.00, 79.00]	68.24 [56.55, 79.56]
Gender, n (%)		
Female	5271 (47.9)	3419 (43.9)
Male	5744 (52.1)	4377 (56.1)
Missing value	1	0
Ethnicity, n (%)		
Asian	177 (1.6)	260 (3.3)
Black/African American	1094 (10.0)	794 (10.2)
Caucasian	8660 (79.0)	5199 (66.7)
Hispanic/Latino	454 (4.1)	290 (3.7)
Native American	86 (0.8)	31 (0.4)
Other/Unknown	485 (4.4)	1222 (15.7)
Missing value	60	0
Admission unit type, n (%)		
Medical intensive care unit (MICU)	1377 (12.5)	2373 (30.4)
Surgical intensive care unit (SICU)	7706 (70.0)	4118 (52.8)
Cardiac related Intensive Care Unit	1682 (15.3)	1164 (14.9)
Neuro-related intensive care unit	251 (2.3)	141 (1.8)
BMI (kg/m^2^), median [IQR]	27.26 [22.96, 33.32]	28.06 [24.07, 33.35]
**Illness severity**		
APS-III, median [IQR]	58.00 [44.00, 76.00]	58.00 [44.00, 79.00]
SOFA, median [IQR]	6.00 [4.00, 8.00]	7.00 [4.00, 10.00]
**Laboratory**		
Platelet count(×109/L), median [IQR]	167.00 [111.00, 234.25]	162.00 [103.00, 235.00]
INR, median [IQR]	1.30 [1.12, 1.70]	1.30 [1.10, 1.60]
Calcium (mg/dL), median [IQR]	7.70 [7.20, 8.30]	7.70 [7.20, 8.30]
BUN (mg/dL), median [IQR]	27.00 [17.00, 44.00]	23.00 [14.00, 39.00]
**Blood gas**		
PaO_2_ (mmHg), median [IQR]	80.00 [64.57, 106.00]	57.00 [39.00, 86.00]
PaCO_2_ (mmHg), median [IQR]	35.00 [29.00, 41.40]	35.00 [30.00, 41.00]
pH, median [IQR]	7.32 [7.23, 7.39]	7.31 [7.23, 7.38]
Base excess (mEq/L), median [IQR]	−4.10 [−9.10, 0.90]	−4.00 [−8.00, 0.00]
**Vital signs**		
Heart rate (/min), median [IQR]	77.00 [66.00, 89.00]	75.00 [64.00, 86.00]
Respiratory rate (/min), median [IQR]	14.00 [11.00, 17.00]	13.00 [11.00, 16.00]
Temperature (°C), median [IQR]	36.40 [36.00, 36.70]	36.44 [36.06, 36.69]
SBP (mmHg), median [IQR]	83.00 [74.00, 95.00]	85.00 [77.00, 94.00]
DBP (mmHg), median [IQR]	44.00 [36.00, 51.00]	43.00 [37.00, 50.00]
**Chronic conditions**		
Charlson comorbidity score, median [IQR]	5.00 [3.00, 7.00]	6.00 [4.00, 8.00]
Chronic pulmonary disease, n (%)		
No	8727 (79.2)	5689 (73.0)
Yes	2289 (20.8)	2107 (27.0)
Diabetes, n (%)		
No	7297 (66.2)	6969 (89.4)
Yes	3719 (33.8)	827 (10.6)
Chronic renal disease, n (%)		
No	8519 (77.3)	5833 (74.8)
Yes	2497 (22.7)	1963 (25.2)
Malignant cancer, n (%)		
No	8992 (81.6)	6392 (82.0)
Yes	2024 (18.4)	1404 (18.0)
**Intervention during ICU**		
Dialysis, n (%)		
No	10 509 (95.4)	7184 (92.1)
Yes	507 (4.6)	612 (7.9)
Invasive mechanical ventilation, n (%)		
No	6928 (62.9)	4067 (52.2)
Yes	4088 (37.1)	3729 (47.8)
Vasopressors, n (%)		
No	6581 (59.7)	6299 (80.8)
Yes	4435 (40.3)	1497 (19.2)
Antiplatelet drugs, n (%)		
No	9112 (82.7)	6754 (86.6)
Yes	1904 (17.3)	1042 (13.4)
**Clinical outcomes**		
ICU length of stay (days), median [IQR]	3.34 [2.10, 5.95]	3.59 [2.04, 7.51]
Hospital length of stay (days), median [IQR]	8.24 [5.12, 13.92]	11.17 [6.37, 20.28]
Clinical outcome, n (%)		
Discharged alive or censored at 28-day	8878 (80.6)	6289 (80.7)
Non-survivors	2138 (19.4)	1507 (19.3)

*BMI* Body Mass Index, *INR* International Normalized Ratio, *BUN* Blood Urea Nitrogen, *SBP* Systolic Blood Pressure, *DBP* Diastolic Blood Pressure, *eICU-CRD* eICU Collaborative Research Database

### Identification of subpopulations using the JLCM

The metrics of the goodness-of-fit model is shown in Table 2 and includes the maximum log-likelihood, AIC, SABIC and entropy for JLCMs, while the optimal number of classes were prespecified from 1 to 6. We observed a consistent decrease in maximum log-likelihood, AIC and SABIC as the number of classes increased. A class of four exhibited the highest entropy, so was considered the best-fitted model, which has maximum log-likelihood of −356,005.9, AIC of 712,079.8 and SABIC of 712,220.2. The average posterior probability of each class is presented in Table S2 (see online supplementary material) and the posterior probabilities above a threshold (%) of each class are presented in Table S3 (see online supplementary material).

**Table 2:** Metrics for determining the optimal number of classes

**No. of classes**	**Log likelihood**	**AIC**	**BIC**	**SABIC**	**Entropy**	**Proportion of people (%)**					
						**Class 1**	**Class 2**	**Class 3**	**Class 4**	**Class 5**	**Class 6**
1	−368180.9	736387.7	736482.7	736441.4	1.000000	100.00					
2	−361110.7	722261.5	722407.6	722344.0	0.288320	41.30	58.70				
3	−357940.8	715935.5	716132.8	716047.0	0.419255	9.69	81.27	9.03			
4	−356005.9	712079.8	712328.3	712220.2	0.500194	6.83	79.44	4.52	9.21		
5	−354668.9	709419.7	709719.3	709589.0	0.477130	4.08	4.31	21.97	4.12	65.51	
6	−353406.6	706909.2	707259.9	707107.4	0.406051	4.14	19.25	42.12	4.31	26.19	3.99

*AIC* akaike information criterion, *BIC* bayesian information criteria, *SABIC* sample-adjusted information criteria

**Figure 2:**
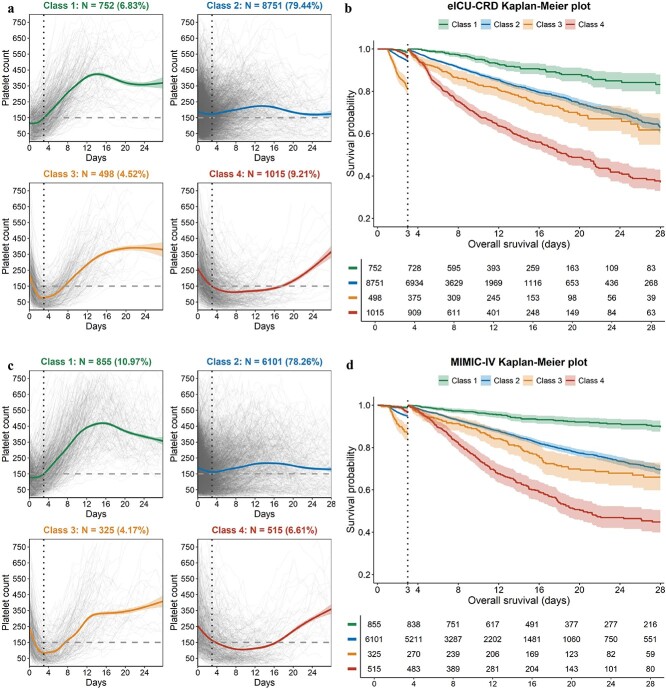
Trajectory plots and Kaplan–Meier survival curves of patients with four dynamic platelet count trajectory patterns. (**a**, **b**) Trajectory plots of platelet count changes within the first 28 days after ICU admission in the eICU-CRD database, along with their corresponding survival curves. (**c**, **d**) Trajectory plots of platelet count changes within the first 28 days after ICU admission in the MIMIC-IV database, along with their corresponding survival curves. *eICU-CRD* eICU Collaborative Research Database

The trajectories are presented in Figure 2a, c. Class 1 is an inverted U shape, while class 2 shows a slight increase. Class 3 exhibited an initial decrease followed by an increase, eventually leveling off. Class 4 showed a rapid decline from a high baseline level and then stabilized at a relatively low level. We labeled the trajectories as follows: class 1 (inverted-U; n = 752; 6.83%), class 2 (slight-increase; n = 8,751; 79.44%), class 3 (decrease-increase-intermediate; n = 498; 4.52%) and class 4 (decrease-low; n = 1015; 9.21%). The average posterior probabilities for the four trajectory groups in eICU-CRD were 0.807 for class 1, 0.689 for class 2, 0.825 for class 3 and 0.797 for class 4 (Table S2, see online supplementary material). Similarly, Tables S4 and S5 (see online supplementary material) provide information on the average posterior probabilities and posterior probabilities above a threshold in the MIMIC-IV.

**Figure 3:**
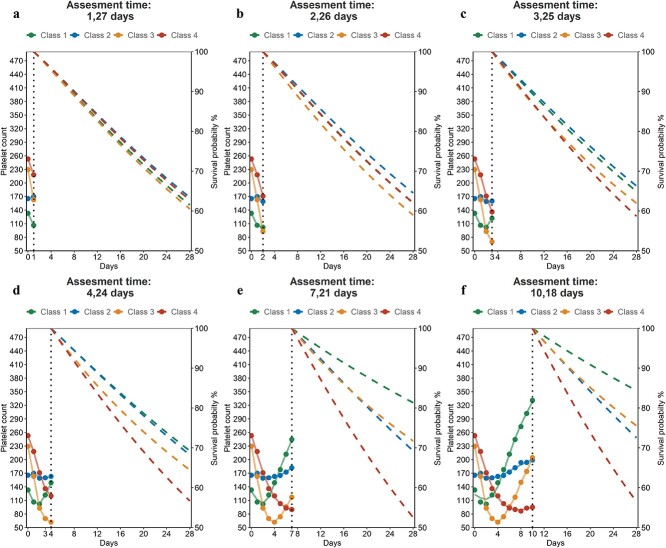
Mean platelet count and predicted survival probability over time for four classes in eICU-CRD. (**a**) Platelet measurements in the first 1 day and predicted survival probability in the next 27 days; (**b**) platelet measurements in the first 2 days and predicted survival probability in the next 26 days; (**c**) platelet measurements in the first 3 days and predicted survival probability in the next 25 days; (**d**) platelet measurements in the first 4 days and predicted survival probability in the next 24 days; (**e**) platelet measurements in the first 7 days and predicted survival probability in the next 21 days; and (**f**) platelet measurements in the first 10 days and predicted survival probability in the next 18 days

We also present the changes in platelet measurements across four classes over different time periods, as well as predicted survival probabilities based on these measurements. We adjusted baseline APS III and CCI for the four groups to ensure consistency. We found that as more platelet measurements were included in the model, the predicted probabilities for the four classes began to diverge. After ~1 week of platelet measurements, the predicted survival probability curves for the four classes were consistent with the actual survival curves (Figure 3).

### Time-dependent HR from piecewise Cox model

The KM curve shows different mortality risk among the four classes in both the eICU-CRD and MIMIC-IV (Figure S1a, b, see online supplementary material). Crossing survival curves indicate potential violation of the proportional hazards (*P* < 0.001). We utilized a piecewise Cox regression to account for time-varying coefficients. We compared the models with different cut points for time splitting, then we selected the model with the highest log-likelihood and determined the optimal cut point of day 3 (Figure S2, see online supplementary material).

The results from the piecewise Cox model showed that class 3 had the highest HR of 13.86 (95% CI: 7.42–25.92) for the first 3 days. From day 3 to day 28, class 4 had the highest risk (HR: 6.09, 95% CI: 4.68–7.92) compared to the other three classes (Table 3). This result was consistent with that from the landmark KM curves (Figure 2a). Patients from class 3 experienced a sharp decline in platelet count in the first 3 days of ICU admission. Patients with a lower baseline platelet count from this class quickly fell below the normal range. From day 3 onwards, the platelet count in class 3 patients started to rise and remained stable above 150 × 10^9^/l, whereas the platelet count in class 4 remained stable at a lower level, slightly below 150 × 10^9^/l. Similar results were observed in the validation set (Figure 2c).

**Table 3:** Time-dependent HR for four classes in the eICU-CRD and MIMIC-IV databases

		**HR (95% CI)**	
	**Time points (day)**	**(0, 3]**	**(3, 28]**
eICU-CRD	Class 2 (slight-increase)	3.90 (2.14, 7.09)	2.32 (1.80, 2.99)
	Class 3 (decrease-increase-intermediate)	13.86 (7.42, 25.92)	2.95 (2.16, 4.03)
	Class 4 (decrease-low)	2.84 (1.46, 5.51)	6.09 (4.68, 7.92)
MIMIC-IV	Class 2 (slight-increase)	4.43 (2.36, 8.32)	3.10 (2.39, 4.03)
	Class 3 (decrease-increase-intermediate)	12.25 (6.17, 24.34)	4.01 (2.87, 5.60)
	Class 4 (decrease-low)	2.83 (1.29, 6.17)	7.84 (5.91, 10.40)

*HR* Hazard ratio, *CI* confidence interval, *eICU-CRD* eICU Collaborative Research Database, *MIMIC-IV* Medical Information Mart for Intensive Care IV

### Clinical characteristics of the four classes

A comparison of baseline characteristics among the four latent classes in two datasets is presented in Tables S6 and S7 (see online supplementary material). In the piecewise Cox model, we found that class 3 and class 4 had the highest risks in both early and later stages. It is noteworthy that although both groups of patients showed a decreasing trend in platelet count after ICU admission, the magnitude of decline differed. We compared the time of onset of thrombocytopenia and severe thrombocytopenia between two groups after ICU admission and found that class 4 patients experienced the aforementioned conditions later than patients from class 3, along with a higher risk of mortality after day 3.

### Subgroup analysis

We conducted a series of analyses stratified by demographics, life support and comorbidities. In both databases, association between trajectory classes and 28-day mortality in septic patients were validated across various strata Figures S3 and S4, see online supplementary material). In the discovery set, we observed a significant interaction between classes and platelet transfusion, with *P*_Interaction_ = 0.035 for class 3 and *P*_Interaction_ = 0.001 for class 4. In the validation set, this interaction was observed only in class 2, with *P*_Interaction_ = 0.013.

### Model evaluation and comparison

In the training set, the dynamic prediction model achieved a C-index of 0.713 (95% CI: 0.702–0.725) at the landmark time of day 1, while the Weibull model yielded a C-index of 0.714 (95% CI: 0.702–0.726). External validation of both models showed similar results, the dynamic prediction model achieved a C-index of 0.739 (95% CI: 0.726–0.751) at day 1 with the Weibull model having a C-index of 0.741 (95% CI: 0.727–0.754).

Both models had C-index values around 0.70 and time-dependent AUC values around 0.60 across the 14 landmark time points. However, as the landmark time increased, there was a gradual decline in these values. Notably, the dynamic prediction model showed a slower decline in AUC values and C-index values over time compared to the Weibull model. This result suggests that when there is a time lag between the start of prediction and the time of variable measurement (e.g. baseline), the dynamic prediction model outperformed the Weibull model in terms of discrimination (Figures S5 and S6, see online supplementary material).

We calculated other model performance metrics, including accuracy, sensitivity and specificity for both models at the 14 landmark time points. The results showed that the dynamic prediction model has better accuracy and specificity (Figure S7–S9, see online supplementary material).

### Individual dynamic prediction

**Figure 4:**
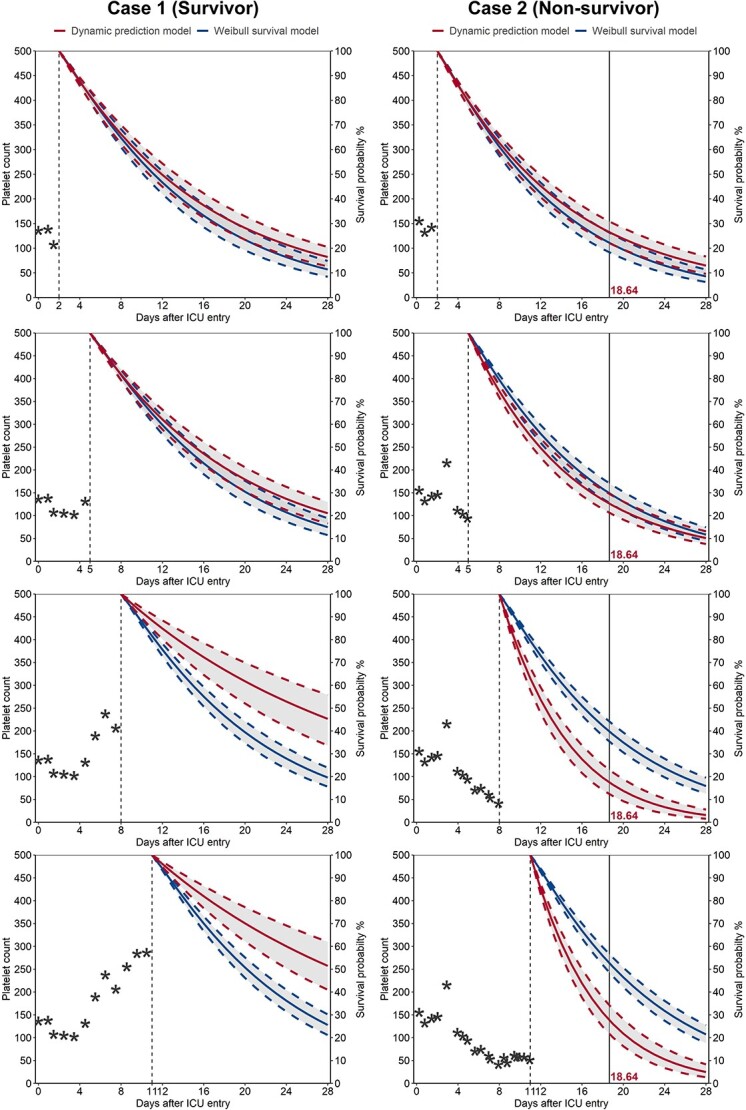
Individual prediction of two selected patients from the MIMIC-IV dataset. On the left is case 1, who stayed alive within 28 days after ICU admission, while on the right is case 2, who experienced death on day 18 of ICU stay. Individual predictions were updated every 3 days from 48 h after ICU admission to day 11. The *x*-axis represents the time since ICU admission, with platelet measurements on the left and model-predicted survival probabilities on the right. The vertical black dashed line indicates the prediction time, and the vertical black solid line represents the time of death

We extracted two patients selected from MIMIC-IV and assessed the performance of individual survival probability predictions of both the dynamic prediction model and the Weibull survival model. The probability of survival was computed every 3 days from 48 h to 14 days after ICU admission (Figure 4). Case 1 is a 55-year-old male who spent 28 days in the ICU without experiencing death. He had an APS III of 160 and a CCI of 3 upon admission, with a baseline platelet count of 136 × 10^9^/l. His platelet count was relatively stable in the first 48 h and started to rise sharply afterwards. Both models returned low 28-day survival probabilities for this patient at 48 h due to his high baseline APS III. However, after including more measures of platelets, the dynamic prediction model was able to increase the survival probability.

Case 2 is a 66-year-old female who died 18 days after ICU admission. She had an APS III score of 86, a CCI of 13 and a baseline platelet count of 132 × 10^9^/l. Her platelet trajectory fluctuated within the first 48 h and steadily declined afterwards, stabilizing at a low level around day 8. Both models returned a low probability of 28-day survival at the 48 h landmark time because of high baseline CCI. The dynamic prediction model takes longitudinal platelet counts into account, therefore providing a more accurate prediction than the Weibull model.

### Sensitivity analysis

Due to limited data on blood culture in the eICU-CRD, we are not able to extract patients strictly following Sepsis-3 [18]. To ensure consistency in patient selection criteria between the two databases, we used the same International Classification of Diseases (ICD) codes for data extraction from both datasets. We also extracted a population from the MIMIC-IV database that adhered to Sepsis-3 for sensitivity analysis and results remained similar (Tables S8 and S9, Figure S10–S12, see online supplementary material).

## Discussion

According to our knowledge, this study is the first to identify heterogenous platelet count trajectories in septic patients and explore the relationship between dynamics in platelet count and in-hospital mortality risk. Additionally, we developed a dynamic prediction model that updates individual survival probability in a real-time manner based on repeated platelet count. Our findings suggest that the dynamic prediction model could capture changes in biomarkers and provide a more accurate prognostic prediction compared to solely considering platelet count at a specific time point.

In this study, we confirmed that the rapid decline of platelet count in the early stage and persistent thrombocytopenia of ICU septic patients are significant prognostic factors [6, 9, 10, 19–23], while septic patients in ICU with increasing platelet count experience more favorable outcomes [11, 24, 25].

Our results are consistent with previous studies that indicate that platelet count reaches a nadir between day 3 and day 4 [9–11, 21, 26] and longer duration of thrombocytopenia is related to a higher mortality rate [9]. In a multi-cohort study involving a mixed ICU population, Chen *et al*. found that the platelet changes in the first 4 days of ICU stay could be classified into three patterns [11]. Among the three trajectory patterns, a decreasing pattern was associated with a higher risk of mortality, while ICU patients with an increase in platelet count had better clinical outcomes. Causes of thrombocytopenia in septic patients vary from decreased production to drug-induced deficiencies [27]. We observed similar characteristics of different classes across two databases. Patients from class 3 had the highest APS III score at baseline, and more patients received invasive mechanical ventilation, vasoactive drugs and blood cells transfusion compared to patients from class 4; however, mortality risk leveled off at a moderate level due to a fast recovery of platelet count. Use of vasopressors and invasive mechanical ventilation have been reported to be associated with higher ICU mortality [28, 29]. However, patients from class 3 responded better to life support than patients from class 4 and experienced fewer days with thrombocytopenia. After 4 days in the ICU, platelet count started to increase in patients in class 3, while most patients in class 4 maintained low platelet count and showed a slow increase thereafter. Notably, the proportion of patients with malignant cancer is the highest in class 4, which encompasses 19.8% of patients in eICU-CRD and 20.8% in MIMIC-IV. Therefore, we speculated on the possibility of drug-induced thrombocytopenia. Chemotherapy with bone marrow inhibition is the most common reason for developing thrombocytopenia in cancer patients [30]. The identification of this subgroup holds significant implications for physicians, offering valuable insights into the underlying causes of thrombocytopenia.

We also observed that class 4 has more patients treated with glucocorticoids. This may because of the multiple putative benefits of glucocorticoids, e.g. there may be modulation of an excess inflammatory response and regulation of adaptive immunity [31]. However, a recent meta-analysis showed that administration of glucocorticoids did not affect mortality in septic patients. Hence, the question of using glucocorticoids in a specific subgroup of patients with sepsis remains open [32].

In summary, multiple studies have reported the prognostic value of platelet count [11, 21]. Our proposed JLCM integrates longitudinal data and survival data by shared latent class, and has the advantage of providing more accurate prediction, especially when the density of the biomarkers is relatively high. Additionally, the JLCM may provide real-time updates of risk probability and thus facilitate precision medicine. Our study provides more evidence of how platelet-count trajectories affect mortality risk, wherein joint modeling is a promising strategy to reveal the potential heterogeneity of septic patients.

We recognize certain limitations in our study. Firstly, this approach often excludes patients who die early after ICU admission and requires a large number of repeated measurements. The characteristics of septic patients differ significantly between the two databases. However, the relatively consistent results from our study further indicate the robustness and the generalizability of the findings.

Secondly, the potential etiology of the platelet count in septic patients was not examined by considering all clinical practices during hospital stay, as it involves multiple complex factors [10, 26, 33]. Therefore, we cannot infer the underlying pathological mechanisms that contribute to the increased mortality rate in patients with a significant decrease in platelet count. Furthermore, our model exhibited a minor disparity in the early predicted survival decline trend within the four potential classes of septic patients, since in cases where there are fewer early platelet measurements or less obvious trends, JLCM may fail to accurately classify these patients and obtain the correct risk function. As an observational study, whether the decrease in platelet count is a cause or a result of the severity of sepsis is unknown. We observed that in certain classes, the subgroup of patients receiving platelet transfusion had a lower risk. However, this result is not entirely consistent across both databases. Improving platelet monitoring and implementing targeted interventions for ICU patients based on current research results require well-designed real-world studies or randomized controlled trials. In addition, when describing the classes identified by JLCM, one should acknowledge that they are derived as non-parametric representations of variation in individual trajectories, rather than just potentially substantive underlying typologies [34].

## Conclusions

In summary, our study highlights the clinical significance of platelet count changes in septic patients after ICU admission. Different platelet count trajectories are associated with mortality risks, with late-stage platelet decrease being a stronger predictor of mortality. Our dynamic prediction model provides real-time updates on risk for septic patients in the ICU, offering valuable practical information for clinical decision-making. These findings have important implications for enhancing clinical management and prognosis prediction in septic patients.

## Abbreviations

AIC: Akaike information criterion; AUC: Area under the receiver operating characteristic curve; APS III: Acute physiology score III; BIC: Bayesian information criteria; C-index: Concordance index; CCI: Charlson comorbidity index; CI: Confidence interval; eICU-CRD: eICU Collaborative Research Database; HR: Hazard ratio; ICU: Intensive care unit; JLCM: Joint latent class model; KM: Kaplan-Meier; MIMIC-IV: Medical Information Mart for Intensive Care IV; SABIC: sample-adjusted Bayesian information criteria.

## Supplementary Material

Supplementary_file_tkae016

## Data Availability

Data are fully available at https://eicu-crd.mit.edu/ and https://mimic.mit.edu/.

## References

[1] Zhang Z , Ho KM, Gu H, Hong Y, Yu Y. Defining persistent critical illness based on growth trajectories in patients with sepsis. Crit Care. 2020;24:57. 32070393 10.1186/s13054-020-2768-z PMC7029548

[2] Mackman N , Tilley RE, Key NS. Role of the extrinsic pathway of blood coagulation in hemostasis and thrombosis. Arterioscler Thromb Vasc Biol. 2007;27:1687–93. 17556654 10.1161/ATVBAHA.107.141911

[3] Clark SR , Ma AC, Tavener SA, McDonald B, Goodarzi Z, Kelly MM, et al. Platelet TLR4 activates neutrophil extracellular traps to ensnare bacteria in septic blood. Nat Med. 2007;13:463–9. 17384648 10.1038/nm1565

[4] de Stoppelaar SF, van 't C, Claushuis TA, Albersen BJ, Roelofs JJ, van der Poll T. Thrombocytopenia impairs host defense in gram-negative pneumonia-derived sepsis in mice. Blood. 2014;124:3781–90. 25301709 10.1182/blood-2014-05-573915 PMC4263985

[5] Claushuis TA , van Vught LA, Scicluna BP, Wiewel MA, Klein Klouwenberg PM, Hoogendijk AJ, et al. Thrombocytopenia is associated with a dysregulated host response in critically ill sepsis patients. Blood. 2016;127:3062–72. 26956172 10.1182/blood-2015-11-680744

[6] Vanderschueren S , De Weerdt A, Malbrain M, Vankersschaever D, Frans E, Wilmer A, et al. Thrombocytopenia and prognosis in intensive care. Crit Care Med. 2000;28:1871–6. 10890635 10.1097/00003246-200006000-00031

[7] Sharma B , Sharma M, Majumder M, Steier W, Sangal A, Kalawar M. Thrombocytopenia in septic shock patients--a prospective observational study of incidence, risk factors and correlation with clinical outcome. Anaesth Intensive Care. 2007;35:874–80. 18084977 10.1177/0310057X0703500604

[8] He J , Wei Y, Chen J, Chen F, Gao W, Lu X. Dynamic trajectory of platelet-related indicators and survival of severe COVID-19 patients. Crit Care. 2020;24:607. 33054834 10.1186/s13054-020-03339-x PMC7556573

[9] Akca S , Haji-Michael P, de Mendonça A, Suter P, Levi M, Vincent JL. Time course of platelet counts in critically ill patients. Crit Care Med. 2002;30:753–6. 11940740 10.1097/00003246-200204000-00005

[10] Greinacher A , Selleng K. Thrombocytopenia in the intensive care unit patient. Hematology Am Soc Hematol Educ Program. 2010;2010:135–43. 21239783 10.1182/asheducation-2010.1.135

[11] Chen J , Gao X, Shen S, Xu J, Sun Z, Lin R, et al. Association of longitudinal platelet count trajectory with ICU mortality: a multi-cohort study. Front Immunol. 2022;13:936662. 10.3389/fimmu.2022.936662. 36059447 PMC9437551

[12] Ghimire S , Ravi S, Budhathoki R, Arjyal L, Hamal S, Bista A, et al. Current understanding and future implications of sepsis-induced thrombocytopenia. Eur J Haematol. 2021;106:301–5. 33191517 10.1111/ejh.13549

[13] Douglas-Louis R , Lou M, Lee B, Minejima E, Bubeck-Wardenburg J, Wong-Beringer A. Prognostic significance of early platelet dynamics in Staphylococcus aureus bacteremia. BMC Infect Dis. 2023;23:82. 36750777 10.1186/s12879-023-08046-w PMC9906934

[14] Kim SY . Determining the number of latent classes in single- and multi-phase growth mixture models. Struct Equ Modeling. 2014;21:263–79. 24729675 10.1080/10705511.2014.882690 PMC3979564

[15] Dekker FW , de Mutsert R, van Dijk PC, Zoccali C, Jager KJ. Survival analysis: time-dependent effects and time-varying risk factors. Kidney Int. 2008;74:994–7. 18633346 10.1038/ki.2008.328

[16] Zhang Z , Reinikainen J, Adeleke KA, Pieterse ME, Groothuis-Oudshoorn CGM. Time-varying covariates and coefficients in cox regression models. Ann Transl Med. 2018;6:121. 29955581 10.21037/atm.2018.02.12 PMC6015946

[17] Proust-Lima C , Taylor JM. Development and validation of a dynamic prognostic tool for prostate cancer recurrence using repeated measures of posttreatment PSA: a joint modeling approach. Biostatistics. 2009;10:535–49. 19369642 10.1093/biostatistics/kxp009 PMC2697347

[18] Singer M , Deutschman CS, Seymour CW, Shankar-Hari M, Annane D, Bauer M, et al. The third international consensus definitions for sepsis and septic shock (Sepsis-3). JAMA. 2016;315:801–10. 26903338 10.1001/jama.2016.0287 PMC4968574

[19] Sprung CL , Peduzzi PN, Shatney CH, Schein RM, Wilson MF, Sheagren JN, et al. Impact of encephalopathy on mortality in the sepsis syndrome. The veterans administration systemic sepsis cooperative study group. Crit Care Med. 1990;18:801–6. 2379391 10.1097/00003246-199008000-00001

[20] Wang LN , He DK, Shao YR, Lv J, Wang PF, Ge Y, et al. Early platelet level reduction as a prognostic factor in intensive care unit patients with severe aspiration pneumonia. Front Physiol. 2023;14:1064699. 36960160 10.3389/fphys.2023.1064699 PMC10029141

[21] Moreau D , Timsit JF, Vesin A, Garrouste-Orgeas M, de Lassence A, Zahar JR, et al. Platelet count decline: an early prognostic marker in critically ill patients with prolonged ICU stays. Chest. 2007;131:1735–41. 17475637 10.1378/chest.06-2233

[22] Kinasewitz GT , Yan SB, Basson B, Comp P, Russell JA, Cariou A, et al. Universal changes in biomarkers of coagulation and inflammation occur in patients with severe sepsis, regardless of causative micro-organism [ISRCTN74215569]. Crit Care. 2004;8:R82–90. 15025782 10.1186/cc2459 PMC420030

[23] Strauss R , Wehler M, Mehler K, Kreutzer D, Koebnick C, Hahn EG. Thrombocytopenia in patients in the medical intensive care unit: bleeding prevalence, transfusion requirements, and outcome. Crit Care Med. 2002;30:1765–71. 12163790 10.1097/00003246-200208000-00015

[24] Zarychanski R , Houston DS. Assessing thrombocytopenia in the intensive care unit: the past, present, and future. Hematology Am Soc Hematol Educ Program. 2017;2017:660–6. 29222318 10.1182/asheducation-2017.1.660 PMC6142536

[25] Nijsten MW , ten Duis HJ, Zijlstra JG, Porte RJ, Zwaveling JH, Paling JC, et al. Blunted rise in platelet count in critically ill patients is associated with worse outcome. Crit Care Med. 2000;28:3843–6. 11153624 10.1097/00003246-200012000-00017

[26] Wang HL , Aguilera C, Knopf KB, Chen TM, Maslove DM, Kuschner WG. Thrombocytopenia in the intensive care unit. J Intensive Care Med. 2013;28:268–80. 22232201 10.1177/0885066611431551

[27] Thiele T , Selleng K, Selleng S, Greinacher A, Bakchoul T. Thrombocytopenia in the intensive care unit-diagnostic approach and management. Semin Hematol. 2013;50:239–50. 23953341 10.1053/j.seminhematol.2013.06.008

[28] Peres Bota D , Melot C, Lopes Ferreira F, Nguyen Ba V, Vincent J-L. The multiple organ dysfunction score (MODS) versus the sequential organ failure assessment (SOFA) score in outcome prediction. Intensive Care Med. 2002;28:1619–24 https://pubmed.ncbi.nlm.nih.gov/12415450. 12415450 10.1007/s00134-002-1491-3

[29] Liu N , Ren J, Yu L, Xie J. Mechanical ventilation associated with worse survival in septic patients: a retrospective analysis of MIMIC-III. J Emerg Crit Care Med. 2020;4. 10.21037/jeccm.2020.01.01.

[30] Gao A , Zhang L, Zhong D. Chemotherapy-induced thrombocytopenia: literature review. Discov Oncol. 2023;14:10. 36695938 10.1007/s12672-023-00616-3 PMC9877263

[31] Cain DW , Cidlowski JA. Immune regulation by glucocorticoids. Nat Rev Immunol. 2017;17:233–47. 28192415 10.1038/nri.2017.1 PMC9761406

[32] Pirracchio R , Annane D, Waschka AK, Lamontagne F, Arabi YM, Bollaert P-E, et al. Patient-level meta-analysis of low-dose hydrocortisone in adults with septic. Shock. 2023;2:EVIDoa2300034. 10.1056/EVIDoa2300034 38320130

[33] Stéphan F , Thiolière B, Verdy E, Tulliez M. Role of hemophagocytic histiocytosis in the etiology of thrombocytopenia in patients with sepsis syndrome or septic shock. Clin Infect Dis. 1997;25:1159–64. 9402376 10.1086/516086

[34] Herle M , Micali N, Abdulkadir M, Loos R, Bryant-Waugh R, Hübel C, et al. Identifying typical trajectories in longitudinal data: modelling strategies and interpretations. Eur J Epidemiol. 2020;35:205–22. 32140937 10.1007/s10654-020-00615-6 PMC7154024

