# Supplementary material for: Serial platelet count as a dynamic prediction marker of hospital mortality among septic patients
Source: Burns Trauma. 2024 Jun 15;12:tkae016. doi: 10.1093/burnst/tkae016 (PMC11179733; doi:10.1093/burnst/tkae016)
Supplement: Supplementary_file_tkae016 [file supplementary_file_tkae016.docx]

Serial Platelet Count as a Dynamic Prediction Marker of Hospital Mortality among Septic Patients

Qian Ye^1†^, Xuan Wang^1†^, Xiaoshuang Xu^1†^, Jiajin Chen^1^, David C. Christiani^2,3^, Feng Chen^1,4,5^, Ruyang Zhang^1*^, Yongyue Wei^1,6*^

^1^Department of Biostatistics, School of Public Health, Center for Global Health, Nanjing Medical University, Nanjing 211166, China

^2^Department of Environmental Health, Harvard T.H. Chan School of Public Health, Harvard University, Boston, MA 02115, USA

^3^Pulmonary and Critical Care Division, Massachusetts General Hospital, Department of Medicine, Harvard Medical School, Boston, MA 02114, USA

^4^Jiangsu Key Lab of Cancer Biomarkers, Prevention and Treatment, Jiangsu Collaborative Innovation Center for Cancer Personalized Medicine, Nanjing Medical University, 211166, Nanjing, China

^5^China International Cooperation Center of Environment and Human Health, Nanjing Medical University

^6^Center for Public Health and Epidemic Preparedness & Response, Peking University, Key Laboratory of Epidemiology of Major Diseases (Peking University), Ministry of Education, 38 Xueyuan Road, Haidian District, Beijing, 100191, China.

†Contributed equally to this work.

*Corresponding authors: Dr. Y.W. ([ywei@pku.edu.cn](mailto:ywei@pku.edu.cn)) or Dr. R.Z. ([zhangruyang@njmu.edu.cn](mailto:zhangruyang@njmu.edu.cn))

Type: Research

Outlines

[Supplementary Methods 4](#_Toc160651628)

[Joint Latent Class Model 4](#_Toc160651629)

[Platelet change patterns 4](#_Toc160651630)

[Dynamic prediction 6](#_Toc160651631)

[Supplementary Tables 7](#_Toc160651632)

[**Supplementary Table 1** Additional baseline characteristics 7](#_Toc160651633)

[**Supplementary Table 2** eICU-CRD posterior classification table 8](#_Toc160651634)

[**Supplementary Table 3** eICU-CRD posterior probabilities above a threshold (%) 9](#_Toc160651635)

[**Supplementary Table 4** MIMIC-IV average posterior probabilities 10](#_Toc160651636)

[**Supplementary Table 5** MIMIC-IV posterior probabilities above a threshold (%) 11](#_Toc160651637)

[**Supplementary Table 6** Baseline characteristics of patients in four latent class in eICU-CRD 12](#_Toc160651638)

[**Supplementary Table 7** Baseline characteristics of patients in four latent class in MIMIC-Ⅳ 16](#_Toc160651639)

[Supplementary Figures 20](#_Toc160651640)

[**Supplementary Figure 1** 28-Day KM survival curves for four classes of septic patients in eICU-CRD and MIMIC-IV databases 20](#_Toc160651641)

[**Supplementary Figure 2** Variations in log-likelihood values of piecewise Cox model with different cut-off points 21](#_Toc160651642)

[**Supplementary Figure 3** Forest plot of subgroup analyses in eICU-CRD 22](#_Toc160651643)

[**Supplementary Figure 4** Forest plot of subgroup analyses in MIMIC-IV 23](#_Toc160651644)

[**Supplementary Figure 5** Time-dependent AUC for two models at different landmark times in two databases 24](#_Toc160651645)

[**Supplementary Figure 6** C-index for two models at different landmark times in two databases 25](#_Toc160651646)

[**Supplementary Figure 7** Accuracy for two models at different landmark times in two databases 26](#_Toc160651647)

[**Supplementary Figure 8** Sensitivity for two models at different landmark times in two databases 27](#_Toc160651648)

[**Supplementary Figure 9** Specificity for two models at different landmark times in two databases 28](#_Toc160651649)

[Sensitive analysis 29](#_Toc160651650)

[**Supplementary Table 8** Baseline characteristics of patients in four latent class in MIMIC-IV Sepsis-3 patients 29](#_Toc160651651)

[**Supplementary Figure 10** Trajectory plots and KM survival curves of patients with four dynamic platelet count trajectory patterns. 33](#_Toc160651652)

[**Supplementary Table 9** Time-dependent HR for four classes in the sensitivity analysis set 34](#_Toc160651653)

[**Supplementary Figure 11** Time-dependent AUC for two models at different landmark in MIMIC-IV Sepsis-3 patients 35](#_Toc160651654)

[**Supplementary Figure 12** C-index for two models at different landmark time in MIMIC-IV Sepsis-3 patients 36](#_Toc160651655)

[References 37](#_Toc160651656)

Supplementary Methods

Joint Latent Class Model

The Joint Latent Class Model (JLCM) is a type of latent class analysis that aims to identify unobserved subgroups of individuals with similar patterns of responses across multiple observed variables .In a joint latent class model (JLCM), the assumption is made that the population consists of heterogeneous subgroups or classes, each with distinct marker trajectories and risks of experiencing an event[1-3].

Platelet change patterns

In this study, we assume heterogeneity in the population of sepsis patients, consisting of G classes of patients. The outcome Y (platelet measurement) for individual *i* at time point *t* conditional on latent class can be represented as follows:

,

,

,

,

.

In our study, we employed a natural spline function with three nodes to capture the shape of the trajectories. No covariates were included to predict class membership.

*Survival model*

For patient *i* belonging to latent class *g*, we can use a proportional hazards model to describe their risk of experiencing a death event at time t:

The is class-specific baseline hazard function in latent class g . For computational convenience, we have used the Weibull hazard function in our study.

To determine the appropriate number of latent classes G, we computed a series of latent class models starting from 1. To identify the model with the optimal number of classes that best fits the data, multiple goodness-of-fit criteria can be compared. The AIC, BIC, and SABIC of the models decreased as the assumed number of latent classes increased, but the differences were small (see Table 2). Therefore, we selected the model with the highest Ramaswamy entropy value as the best latent class model[4-6]. The R package *lcmm* (version 2.0.2) was used for the Joint latent class modeling.

We employed a Cox hazard model with time-dependent coefficients to further explore the differential effects of various class on patient prognosis. The model is an extension of the conventional Cox proportional hazard model, allowing the coefficients to vary over time[7]:

This is a single-variable piecewise proportional hazards (PWPH) model with a single cut point at t = 3 days. PWPH models refer to regression models that exhibit proportional hazards characteristics when constrained within different time intervals. Here, *λ₀*(*t*) represents the baseline hazard function, while *α* and *β* represent the coefficients for the latent class when follow-up time is ≤ 3 days and > 3 days, respectively, reflecting the time-dependent effects of covariates. This allowed us to explore how the effects of different platelet patterns change over time.

Dynamic prediction

The JLCM allows for the computation of posterior probabilities of event occurrence within a certain time window based on the available data of a patient at the current time. These probabilities, similar to survival models, can be used to predict the risk of an event happening for a patient at a specified time. The calculation of the posterior probability of an event occurrence for subject *i* in a specified time window [*s*, *s* + *t*] for a patient in the JLCM is as follows:

In the above equation, *s* is the prediction time (*s* ≥ 0) and *t* is the horizon (*t* ≥ 0), is the vector of repeated PLT measures until s and are all the covariates.

Probability of death in Weibull Survival model

In the Weibull survival model, the calculation of the probability of a patient experiencing a death event during the time period [*s*, *s* + *t*], based on their baseline covariates and baseline platelet measurement, is as follows:

,

where *k* is the shape parameter, *λ* is the scale parameter, and *s* and *t* are as defined before.

Supplementary Tables

**Table S1.** Additional baseline characteristics

|  | **eICU-CRD v2.0 (N = 11,016)** | **MIMIC-IV v2.0 (N = 7,796)** |
| --- | --- | --- |
| Basic Characteristics |  |  |
| Weight (kg), median (IQR) | 78.00 [64.40, 96.02] | 79.17 [65.90, 95.74] |
| Height (cm), median (IQR) | 168.00 [160.00, 177.80] | 170.00 [160.00, 178.00] |
| Laboratory |  |  |
| White Blood Cell(×10^9^/L), median (IQR) | 11.81 [7.70, 17.10] | 10.30 [6.70, 14.90] |
| Hematocrit (%), median (IQR) | 30.55 [26.10, 35.00] | 29.00 [24.70, 33.60] |
| Bilirubin (mg/dL), median (IQR) | 0.70 [0.40, 1.20] | 0.80 [0.40, 1.80] |
| Creatinine (mg/dL), median (IQR) | 1.30 [0.86, 2.21] | 1.10 [0.70, 1.80] |
| Blood Gas |  |  |
| PaO_2_ (mmHg), median (IQR) | 80.00 [64.57, 106.00] | 57.00 [39.00, 86.00] |
| PaCO_2_ (mmHg), median (IQR) | 35.00 [29.00, 41.40] | 35.00 [30.00, 41.00] |
| pH, median (IQR) | 7.32 [7.23, 7.39] | 7.31 [7.23, 7.38] |
| Vital Signs |  |  |
| Heart Rate (/min), median (IQR) | 77.00 [66.00, 89.00] | 75.00 [64.00, 86.00] |
| Respiratory Rate (/min), median (IQR) | 14.00 [11.00, 17.00] | 13.00 [11.00, 16.00] |
| Temperature (℃), median (IQR) | 36.40 [36.00, 36.70] | 36.44 [36.06, 36.69] |
| DBP (mmHg), median (IQR) | 44.00 [36.00, 51.00] | 43.00 [37.00, 50.00] |
| Intervention during ICU |  |  |
| Antibiotics, n (%) |  |  |
| No | 2836 (25.7) | 2499 (32.1) |
| Yes | 8180 (74.3) | 5297 (67.9) |
| Glucocorticoids, n (%) |  |  |
| No | 8837 (80.2) | 7389 (94.8) |
| Yes | 2179 (19.8) | 407 (5.2) |
| Blood Transfusion, n (%) |  |  |
| No | 8403 (76.3) | 5847 (75.0) |
| Yes | 2613 (23.7) | 1949 (25.0) |
| Platelet Transfusion, n (%) |  |  |
| No | 10505 (95.4) | 7165 (91.9) |
| Yes | 511 (4.6) | 631 (8.1) |

**Table S2.** eICU-CRD posterior classification table

|  | **prob1** | **prob2** | **prob3** | **prob4** |
| --- | --- | --- | --- | --- |
| Class1 | 0.825 | 0.164 | 0.008 | 0.003 |
| Class2 | 0.161 | 0.689 | 0.021 | 0.129 |
| Class3 | 0.029 | 0.085 | 0.807 | 0.080 |
| Class4 | 0.006 | 0.139 | 0.059 | 0.797 |

Each patient can calculate their posterior probabilities for belonging to each of the four classes. The numbers in the table represent the average posterior probabilities (Prob1, Prob2, Prob3, Prob4) of patients in each class (Class1, Class2, Class3, Class4) in the eICU-CRD.

**Table S3.** eICU-CRD posterior probabilities above a threshold (%)

|  | **Class1** | **Class2** | **Class3** | **Class4** |
| --- | --- | --- | --- | --- |
| prob>0.5 | 96.14 | 95.17 | 90.16 | 89.46 |
| prob>0.7 | 69.28 | 33.50 | 68.07 | 64.04 |
| prob>0.9 | 51.33 | 14.02 | 47.79 | 46.40 |

The proportion of patients in each class (Class1, Class2, Class3, Class4) whose posterior probability of belonging to that class is greater than the specified threshold (0.5, 0.7, 0.9) in the eICU-CRD.

**Table S4.** MIMIC-IV average posterior probabilities

|  | **prob1** | **prob2** | **prob3** | **prob4** |
| --- | --- | --- | --- | --- |
| Class1 | 0.832 | 0.156 | 0.007 | 0.004 |
| Class2 | 0.191 | 0.694 | 0.019 | 0.096 |
| Class3 | 0.016 | 0.060 | 0.852 | 0.072 |
| Class4 | 0.006 | 0.107 | 0.05 | 0.837 |

Each patient can calculate their posterior probabilities for belonging to each of the four classes. The numbers in the table represent the average posterior probabilities (Prob1, Prob2, Prob3, Prob4) of patients in each class (Class1, Class2, Class3, Class4) in MIMIC-IV database.

**Table S5.** MIMIC-IV posterior probabilities above a threshold (%)

|  | **Class1** | **Class2** | **Class3** | **Class4** |
| --- | --- | --- | --- | --- |
| prob>0.5 | 95.20 | 95.53 | 91.69 | 90.10 |
| prob>0.7 | 68.77 | 32.19 | 76.62 | 72.23 |
| prob>0.9 | 56.37 | 19.60 | 59.38 | 57.86 |

The proportion of patients in each class (Class1, Class2, Class3, Class4) whose posterior probability of belonging to that class is greater than the specified threshold (0.5, 0.7, 0.9) in the MIMIC-IV database.

**Table S6.** Baseline characteristics of patients in four latent class in eICU-CRD

|  | Class1 (N = 752) | Class2 (N = 8,751) | Class3 (N = 498) | Class4 (N = 1,015) | P |
| --- | --- | --- | --- | --- | --- |
| Basic Characteristics |  |  |  |  |  |
| Age (years), median (IQR) | 61.00 [49.00, 73.00] | 68.00 [57.00, 79.00] | 64.00 [55.00, 75.00] | 68.00 [59.00, 77.00] | <0.001 |
| Gender, n (%) |  |  |  |  |  |
| Female | 328 (43.6) | 4150 (47.4) | 276 (55.4) | 517 (50.9) | <0.001 |
| Male | 424 (56.4) | 4600 (52.6) | 222 (44.6) | 498 (49.1) |  |
| Ethnicity, n (%) |  |  |  |  |  |
| Asian | 14 (1.9) | 144 (1.7) | 4 (0.8) | 15 (1.5) | 0.815 |
| Black/African American | 78 (10.5) | 873 (10.0) | 40 (8.1) | 103 (10.2) |  |
| Caucasian | 581 (78.0) | 6890 (79.1) | 400 (80.6) | 789 (78.4) |  |
| Hispanic/Latino | 31 (4.2) | 353 (4.1) | 25 (5.0) | 45 (4.5) |  |
| Native American | 9 (1.2) | 63 (0.7) | 3 (0.6) | 11 (1.1) |  |
| Other/Unknown | 32 (4.3) | 385 (4.4) | 24 (4.8) | 44 (4.4) |  |
| Admission Unit Type, n (%) |  |  |  |  |  |
| Medical Intensive Care Unit (MICU) | 1067 (12.5) | 52 (10.5) | 169 (12.5) | 89 (13.4) | 0.592 |
| Surgical Intensive Care Unit (SICU) | 5960 (70.1) | 362 (73.1) | 939 (69.5) | 445 (67.1) |  |
| Cardiac related Intensive Care Unit | 1279 (15.0) | 73 (14.7) | 216 (16.0) | 114 (17.2) |  |
| Neuro related Intensive Care Unit | 200 (2.4) | 8 (1.6) | 28 (2.1) | 15 (2.3) |  |
| BMI (kg/m^2^), median (IQR) | 27.46 [23.50, 32.93] | 27.34 [23.00, 33.45] | 26.78 [22.52, 32.21] | 26.63 [22.46, 32.90] | 0.026 |
| Illness Severity |  |  |  |  |  |
| APS-Ⅲ, median (IQR) | 63.00 [49.00, 79.00] | 55.00 [42.00, 71.00] | 82.00 [63.00, 106.75] | 72.00 [56.00, 91.00] | <0.001 |
| SOFA, median (IQR) | 7.00 [5.00, 9.00] | 6.00 [4.00, 8.00] | 9.00 [6.00, 11.00] | 8.00 [5.00, 10.00] | <0.001 |
| Laboratory |  |  |  |  |  |
| Platelet Count(×109/L), median (IQR) | 111.00 [59.00, 166.00] | 169.00 [115.00, 234.00] | 148.50 [95.50, 216.00] | 208.00 [142.00, 305.00] | <0.001 |
| INR, median (IQR) | 1.30 [1.10, 1.59] | 1.30 [1.10, 1.60] | 1.46 [1.20, 1.71] | 1.40 [1.20, 1.90] | <0.001 |
| Calcium (mg/dL), median (IQR) | 7.50 [6.90, 8.00] | 7.80 [7.30, 8.30] | 7.00 [6.30, 7.60] | 7.60 [6.90, 8.20] | <0.001 |
| BUN (mg/dL), median (IQR) | 23.00 [14.00, 39.00] | 27.00 [17.00, 42.00] | 32.00 [20.00, 48.00] | 33.00 [20.00, 55.00] | <0.001 |
| Blood Gas |  |  |  |  |  |
| PaO_2_ (mmHg), median (IQR) | 78.00 [64.00, 103.00] | 81.00 [65.70, 107.50] | 75.00 [59.72, 99.25] | 77.00 [63.00, 104.00] | <0.001 |
| PaCO_2_ (mmHg), median (IQR) | 34.00 [28.63, 39.75] | 35.80 [30.00, 42.00] | 31.00 [25.50, 37.95] | 33.00 [27.17, 40.00] | <0.001 |
| pH, median (IQR) | 7.33 [7.25, 7.40] | 7.34 [7.26, 7.40] | 7.21 [7.10, 7.31] | 7.29 [7.18, 7.37] | <0.001 |
| Base Excess (mEq/L), median (IQR) | -5.00 [-8.70, 0.00] | -3.00 [-8.00, 1.00] | -11.95 [-16.20, -6.93] | -6.80 [-12.70, 0.00] | <0.001 |
| Vital Signs |  |  |  |  |  |
| Heart Rate (/min), median (IQR) | 80.00 [69.00, 93.00] | 76.00 [65.00, 88.00] | 85.00 [72.00, 98.00] | 80.00 [69.00, 94.00] | <0.001 |
| Respiratory Rate (/min), median (IQR) | 15.00 [12.00, 18.00] | 14.00 [11.00, 17.00] | 14.00 [11.00, 17.00] | 14.00 [11.00, 18.00] | 0.003 |
| Temperature (℃), median (IQR) | 36.60 [36.20, 36.90] | 36.40 [36.10, 36.70] | 36.20 [35.60, 36.70] | 36.30 [35.70, 36.60] | <0.001 |
| SBP (mmHg), median (IQR) | 84.00 [75.00, 97.00] | 84.00 [75.00, 96.00] | 75.00 [65.00, 85.00] | 78.00 [68.00, 90.00] | <0.001 |
| DBP (mmHg), median (IQR) | 46.00 [39.00, 54.00] | 45.00 [37.00, 52.00] | 39.00 [30.00, 47.00] | 41.00 [33.00, 49.00] | <0.001 |
| Chronic Conditions |  |  |  |  |  |
| Charlson Comorbidity Score, median (IQR) | 4.00 [2.00, 6.00] | 5.00 [3.00, 7.00] | 4.00 [3.00, 6.00] | 5.00 [4.00, 7.00] | <0.001 |
| Chronic Pulmonary Disease, n (%) |  |  |  |  |  |
| No | 647 (86.0) | 6889 (78.7) | 404 (81.1) | 787 (77.5) | <0.001 |
| Yes | 105 (14.0) | 1862 (21.3) | 94 (18.9) | 228 (22.5) |  |
| Diabetes, n (%) |  |  |  |  |  |
| No | 560 (74.5) | 5729 (65.5) | 343 (68.9) | 665 (65.5) | <0.001 |
| Yes | 192 (25.5) | 3022 (34.5) | 155 (31.1) | 350 (34.5) |  |
| Chronic Renal Disease, n (%) |  |  |  |  |  |
| No | 624 (83.0) | 6745 (77.1) | 412 (82.7) | 738 (72.7) | <0.001 |
| Yes | 128 (17.0) | 2006 (22.9) | 86 (17.3) | 277 (27.3) |  |
| Malignant Cancer, n (%) |  |  |  |  |  |
| No | 620 (82.4) | 7133 (81.5) | 425 (85.3) | 814 (80.2) | 0.094 |
| Yes | 132 (17.6) | 1618 (18.5) | 73 (14.7) | 201 (19.8) |  |
| Intervention during ICU |  |  |  |  |  |
| Dialysis, n (%) |  |  |  |  |  |
| No | 740 (98.4) | 8340 (95.3) | 483 (97.0) | 946 (93.2) | <0.001 |
| Yes | 12 (1.6) | 411 (4.7) | 15 (3.0) | 69 (6.8) |  |
| Invasive Mechanical Ventilation, n (%) |  |  |  |  |  |
| No | 346 (46.0) | 5954 (68.0) | 145 (29.1) | 483 (47.6) | <0.001 |
| Yes | 406 (54.0) | 2797 (32.0) | 353 (70.9) | 532 (52.4) |  |
| Vasopressors, n (%) |  |  |  |  |  |
| No | 470 (62.5) | 5576 (63.7) | 153 (30.7) | 382 (37.6) | <0.001 |
| Yes | 282 (37.5) | 3175 (36.3) | 345 (69.3) | 633 (62.4) |  |
| Antiplatelet Drugs, n (%) |  |  |  |  |  |
| No | 646 (85.9) | 7240 (82.7) | 418 (83.9) | 808 (79.6) | 0.005 |
| Yes | 106 (14.1) | 1511 (17.3) | 80 (16.1) | 207 (20.4) |  |
| Antibiotics, n (%) |  |  |  |  |  |
| No | 174 (23.1) | 2261 (25.8) | 134 (26.9) | 267 (26.3) | 0.357 |
| Yes | 578 (76.9) | 6490 (74.2) | 364 (73.1) | 748 (73.7) |  |
| Glucocorticoids, n (%) |  |  |  |  |  |
| No | 613 (81.5) | 7111 (81.3) | 371 (74.5) | 742 (73.1) | <0.001 |
| Yes | 139 (18.5) | 1640 (18.7) | 127 (25.5) | 273 (26.9) |  |
| Blood Transfusion, n (%) |  |  |  |  |  |
| No | 530 (70.5) | 6800 (77.7) | 338 (67.9) | 735 (72.4) | <0.001 |
| Yes | 222 (29.5) | 1951 (22.3) | 160 (32.1) | 280 (27.6) |  |
| Platelet Transfusion, n (%) |  |  |  |  |  |
| No | 679 (90.3) | 8449 (96.5) | 444 (89.2) | 933 (91.9) | <0.001 |
| Yes | 73 (9.7) | 302 (3.5) | 54 (10.8) | 82 (8.1) |  |
| Clinical Outcomes |  |  |  |  |  |
| Thrombocytopenia time^1^ | 0.26 [0.08, 0.58] | 0.41 [0.16, 0.85] | 0.87 [0.31, 1.58] | 1.70 [0.55, 3.23] | <0.001 |
| Severe Thrombocytopenia time^2^ | 0.42 [0.16, 0.76] | 0.91 [0.32, 1.67] | 2.43 [1.73, 3.08] | 4.37 [2.78, 5.95] | <0.001 |
| ICU length of stay (days), median (IQR) | 7.18 [4.83, 10.62] | 2.88 [1.96, 4.66] | 7.54 [3.12, 12.44] | 6.14 [4.46, 9.70] | <0.001 |
| Hospital length of stay (days), median (IQR) | 13.75 [9.30, 20.17] | 7.63 [4.86, 12.33] | 12.63 [5.51, 18.92] | 11.03 [6.66, 17.49] | <0.001 |
| Clinical outcome, n (%) |  |  |  |  |  |
| Discharged alive or censored at 28-day | 677 (90.0) | 7332 (83.8) | 303 (60.8) | 566 (55.8) | <0.001 |
| Non-survivors | 75 (10.0) | 1419 (16.2) | 195 (39.2) | 449 (44.2) |  |

1: Thrombocytopenia time is defined as the time at which a patient's platelet measurement falls below 150×10^9 /L for the first time; 2: Severe Thrombocytopenia time is defined as the time at which a patient's platelet measurement falls below 20×10^9 /L for the first time.

**Table S7.** Baseline characteristics of patients in four latent class in MIMIC-Ⅳ

|  | Class1 (N=855) | Class2 (N=6,101) | Class3 (N=325) | Class4 (N=515) | P |
| --- | --- | --- | --- | --- | --- |
| Basic Characteristics |  |  |  |  |  |
| Age (years), median (IQR) | 61.68 [48.19,73.25] | 69.31 [57.41,80.51] | 65.30 [54.91,75.29] | 68.81 [58.76,78.12] | <0.001 |
| Gender, n (%) |  |  |  |  |  |
| Female | 341 (39.9) | 2683 (44.0) | 164 (50.5) | 231 (44.9) | 0.009 |
| Male | 514 (60.1) | 3418 (56.0) | 161 (49.5) | 284 (55.1) |  |
| Ethnicity, n (%) |  |  |  |  |  |
| Asian | 22 (2.6) | 213 (3.5) | 8 (2.5) | 17 (3.3) | 0.001 |
| Black/African American | 78 (9.1) | 650 (10.7) | 20 (6.2) | 46 (8.9) |  |
| Caucasian | 563 (65.8) | 4094 (67.1) | 220 (67.7) | 322 (62.5) |  |
| Hispanic/Latino | 30 (3.5) | 225 (3.7) | 13 (4.0) | 22 (4.3) |  |
| Native American | 0 (0.0) | 28 (0.5) | 1 (0.3) | 2 (0.4) |  |
| Other/Unknown | 162 (18.9) | 891 (14.6) | 63 (19.4) | 106 (20.6) |  |
| Admission Unit Type, n (%) |  |  |  |  |  |
| Medical Intensive Care Unit (MICU) | 1801 (31.6) | 92 (20.9) | 388 (28.8) | 92 (30.3) | <0.001 |
| Surgical Intensive Care Unit (SICU) | 2953 (51.8) | 246 (55.8) | 766 (56.8) | 153 (50.3) |  |
| Cardiac related Intensive Care Unit | 846 (14.8) | 98 (22.2) | 165 (12.2) | 55 (18.1) |  |
| Neuro related Intensive Care Unit | 102 (1.8) | 5 (1.1) | 30 (2.2) | 4 (1.3) |  |
| BMI (kg/m^2^), median (IQR) | 28.43 [24.60,33.31] | 27.92 [23.97,33.28] | 27.90 [24.29,33.85] | 28.25 [24.06,33.48] | 0.612 |
| Illness Severity |  |  |  |  |  |
| APS-Ⅲ, median (IQR) | 68.00 [51.00,87.00] | 54.00 [41.00,72.00] | 86.00 [65.00,109.00] | 80.00 [64.00,104.00] | <0.001 |
| SOFA, median (IQR) | 9.00 [6.00,12.00] | 6.00 [4.00,10.00] | 12.00 [8.00,14.00] | 9.00 [7.00,13.00] | <0.001 |
| Laboratory |  |  |  |  |  |
| Platelet Count(×10^9^/L), median (IQR) | 112.00 [53.50,171.00] | 167.00 [108.00,239.00] | 157.00 [100.00,232.00] | 197.00 [125.50,302.50] | <0.001 |
| INR, median (IQR) | 1.20 [1.10,1.40] | 1.30 [1.10,1.60] | 1.30 [1.10,1.60] | 1.40 [1.20,1.80] | <0.001 |
| Calcium (mg/dL), median (IQR) | 7.50 [6.90,8.00] | 7.80 [7.30,8.40] | 7.30 [6.70,7.90] | 7.60 [7.10,8.30] | <0.001 |
| BUN (mg/dL), median (IQR) | 21.00 [13.00,36.00] | 23.00 [15.00,39.00] | 25.00 [16.00,39.00] | 30.00 [18.00,53.00] | <0.001 |
| Blood Gas |  |  |  |  |  |
| PaO_2_ (mmHg), median (IQR) | 60.00 [41.00,84.00] | 57.00 [38.00,86.00] | 54.00 [39.00,80.25] | 56.00 [38.00,82.00] | 0.326 |
| PaCO_2_ (mmHg), median (IQR) | 34.00 [30.00,39.00] | 35.00 [30.00,41.00] | 31.00 [26.00,35.00] | 33.00 [28.00,39.00] | <0.001 |
| pH, median (IQR) | 7.29 [7.20,7.37] | 7.32 [7.25,7.38] | 7.19 [7.10,7.27] | 7.28 [7.19,7.36] | <0.001 |
| Base Excess (mEq/L), median (IQR) | -5.00 [-9.00,-1.00] | -3.00 [-7.00,0.00] | -11.00 [-15.00,-6.00] | -6.00 [-10.00,-1.00] | <0.001 |
| Vital Signs |  |  |  |  |  |
| Heart Rate (/min), median (IQR) | 79.00 [67.00,92.00] | 74.00 [63.00,85.00] | 82.00 [68.00,92.25] | 75.00 [64.00,89.00] | <0.001 |
| Respiratory Rate (/min), median (IQR) | 13.00 [11.00,16.00] | 13.00 [11.00,16.00] | 13.00 [10.00,16.00] | 13.00 [11.00,16.00] | 0.122 |
| Temperature (℃), median (IQR) | 36.50 [36.17,36.83] | 36.44 [36.11,36.67] | 36.39 [35.50,36.67] | 36.33 [35.67,36.61] | <0.001 |
| SBP (mmHg), median (IQR) | 85.00 [76.00,93.00] | 86.00 [78.00,95.00] | 79.00 [68.75,87.00] | 82.00 [74.00,90.00] | <0.001 |
| DBP (mmHg), median (IQR) | 44.00 [39.00,51.00] | 43.50 [37.00,50.00] | 41.00 [34.00,47.00] | 42.00 [35.00,48.00] | <0.001 |
| Chronic Conditions |  |  |  |  |  |
| Charlson Comorbidity Score, median (IQR) | 5.00 [3.00,7.00] | 6.00 [5.00,8.00] | 6.00 [4.00,8.00] | 7.00 [5.00,9.00] | <0.001 |
| Chronic Pulmonary Disease, n (%) |  |  |  |  |  |
| No | 657 (76.8) | 4433 (72.7) | 233 (71.7) | 366 (71.1) | 0.046 |
| Yes | 198 (23.2) | 1668 (27.3) | 92 (28.3) | 149 (28.9) |  |
| Diabetes, n (%) |  |  |  |  |  |
| No | 793 (92.7) | 5409 (88.7) | 302 (92.9) | 465 (90.3) | <0.001 |
| Yes | 62 (7.3) | 692 (11.3) | 23 (7.1) | 50 (9.7) |  |
| Chronic Renal Disease, n (%) |  |  |  |  |  |
| No | 720 (84.2) | 4475 (73.3) | 261 (80.3) | 377 (73.2) | <0.001 |
| Yes | 135 (15.8) | 1626 (26.7) | 64 (19.7) | 138 (26.8) |  |
| Malignant Cancer, n (%) |  |  |  |  |  |
| No | 713 (83.4) | 5002 (82.0) | 269 (82.8) | 408 (79.2) | 0.268 |
| Yes | 142 (16.6) | 1099 (18.0) | 56 (17.2) | 107 (20.8) |  |
| Intervention during ICU |  |  |  |  |  |
| Dialysis, n (%) |  |  |  |  |  |
| No | 802 (93.8) | 5673 (93.0) | 264 (81.2) | 445 (86.4) | <0.001 |
| Yes | 53 (6.2) | 428 (7.0) | 61 (18.8) | 70 (13.6) |  |
| Invasive Mechanical Ventilation, n (%) |  |  |  |  |  |
| No | 259 (30.3) | 3633 (59.5) | 64 (19.7) | 111 (21.6) | <0.001 |
| Yes | 596 (69.7) | 2468 (40.5) | 261 (80.3) | 404 (78.4) |  |
| Vasopressors, n (%) |  |  |  |  |  |
| No | 651 (76.1) | 5247 (86.0) | 140 (43.1) | 261 (50.7) | <0.001 |
| Yes | 204 (23.9) | 854 (14.0) | 185 (56.9) | 254 (49.3) |  |
| Antiplatelet Drugs, n (%) |  |  |  |  |  |
| No | 749 (87.6) | 5348 (87.7) | 259 (79.7) | 398 (77.3) | <0.001 |
| Yes | 106 (12.4) | 753 (12.3) | 66 (20.3) | 117 (22.7) |  |
| Antibiotics, n (%) |  |  |  |  |  |
| No | 127 (14.9) | 2293 (37.6) | 43 (13.2) | 36 (7.0) | <0.001 |
| Yes | 728 (85.1) | 3808 (62.4) | 282 (86.8) | 479 (93.0) |  |
| Glucocorticoids, n (%) |  |  |  |  |  |
| No | 803 (93.9) | 5809 (95.2) | 310 (95.4) | 467 (90.7) | <0.001 |
| Yes | 52 (6.1) | 292 (4.8) | 15 (4.6) | 48 (9.3) |  |
| Blood Transfusion, n (%) |  |  |  |  |  |
| No | 543 (63.5) | 4951 (81.2) | 136 (41.8) | 217 (42.1) | <0.001 |
| Yes | 312 (36.5) | 1150 (18.8) | 189 (58.2) | 298 (57.9) |  |
| Platelet Transfusion, n (%) |  |  |  |  |  |
| No | 763 (89.2) | 5751 (94.3) | 248 (76.3) | 403 (78.3) | <0.001 |
| Yes | 92 (10.8) | 350 (5.7) | 77 (23.7) | 112 (21.7) |  |
| Clinical Outcomes |  |  |  |  |  |
| Thrombocytopenia time^1^ | 0.13 [0.04, 0.34] | 0.18 [0.06, 0.58] | 0.86 [0.29, 1.67] | 1.25 [0.22, 3.45] | <0.001 |
| Severe Thrombocytopenia time^2^ | 0.18 [0.05, 0.72] | 1.35 [0.41, 3.19] | 2.57 [1.74, 4.08] | 6.11 [3.55, 7.89] | <0.001 |
| ICU length of stay (days), median (IQR) | 7.64 [4.62,13.59] | 2.92 [1.90,5.38] | 9.00 [3.83,14.92] | 8.67 [5.31,13.59] | <0.001 |
| Hospital length of stay (days), median (IQR) | 19.02 [11.94,29.65] | 9.80 [5.83,17.68] | 17.40 [8.62,25.49] | 15.01 [9.50,24.67] | <0.001 |
| Clinical outcome, n (%) |  |  |  |  |  |
| Discharged alive or censored at 28-day | 784 (91.7) | 5031 (82.5) | 202 (62.2) | 272 (52.8) | <0.001 |
| Non-survivors | 71 (8.3) | 1070 (17.5) | 123 (37.8) | 243 (47.2) |  |

1: Thrombocytopenia time is defined as the time at which a patient's platelet measurement falls below 150×10^9 /L for the first time; 2: Severe Thrombocytopenia time is defined as the time at which a patient's platelet measurement falls below 20×10^9 /L for the first time.

Supplementary Figures

**Figure S1.** 28-Day KM survival curves for four classes of septic patients in eICU-CRD and MIMIC-IV databases

The KM survival curves for 28-day survival of septic patients with four different platelet count trajectory patterns in the ICU.

**Figure S2.** Variations in log-likelihood values of piecewise Cox model with different cut-off points

The graph displays the variations in the log-likelihood values of the piecewise Cox model when setting continuous cut-off points within 28 days.

**Figure S3.** Forest plot of subgroup analyses in eICU-CRD


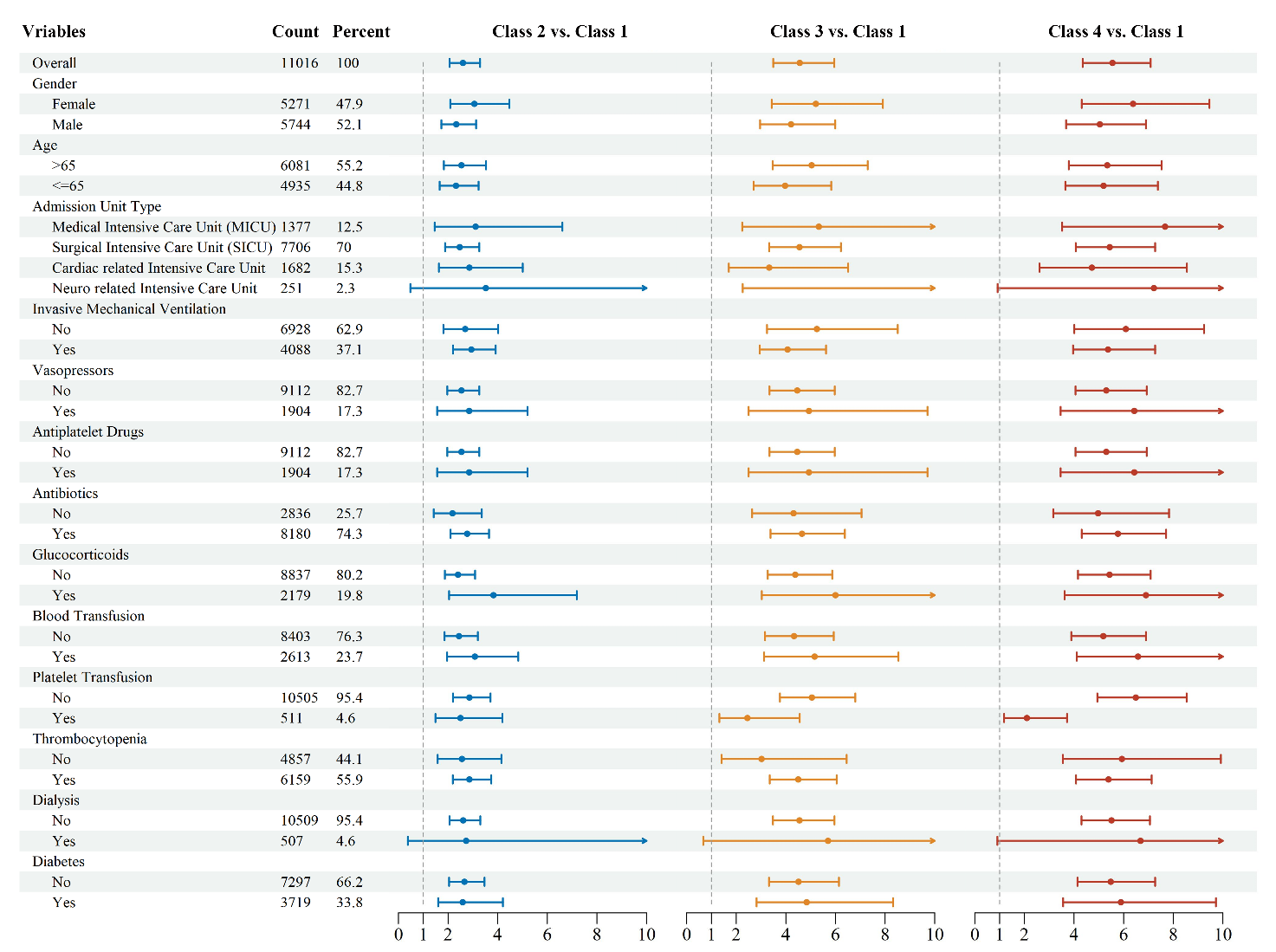


The horizontal axis in the figure represents the hazard ratio (HR), with Class 1 as the reference. The dots in the forest plot represent point estimates for HR, and the lines indicate the corresponding 95% confidence intervals.

**Figure S4.** Forest plot of subgroup analyses in MIMIC-IV


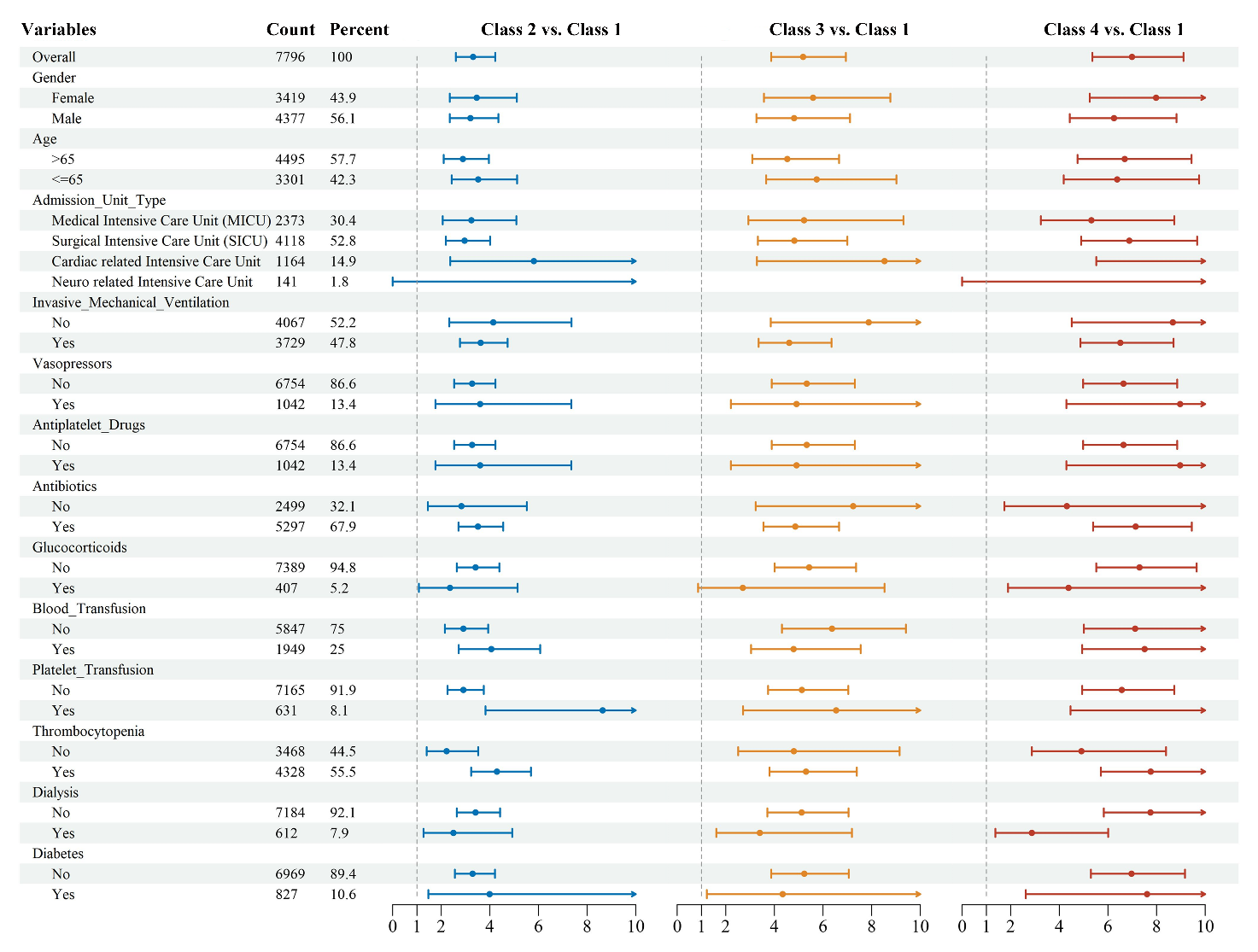


The horizontal axis in the figure represents the hazard ratio (HR), with Class 1 as the reference. The dots in the forest plot represent point estimates for HR, and the lines indicate the corresponding 95% confidence intervals.

**Figure S5.** Time-dependent AUC for two models at different landmark times in two databases

The numbers in the plot represent the p-values of the time-dependent AUC differences between two models, calculated through permutation tests by shuffling 2000 times at each landmark time.

**Figure S6.** C-index for two models at different landmark times in two databases

The numbers in the plot represent the p-values of the C-index differences between two models, calculated through permutation tests by shuffling 2000 times at each landmark time.

**Figure S7.** Accuracy for two models at different landmark times in two databases

The Scatter plot illustrates the variations in accuracy (ACC) over time for two models. The results are presented for different prediction start times from the eICU-CRD and MIMIC-IV.

**Figure S8.** Sensitivity for two models at different landmark times in two databases

The Scatter plot illustrates the variations in sensitivity over time for two models. The results are presented for different prediction start times from the eICU-CRD and MIMIC-IV.

**Figure S9.** Specificity for two models at different landmark times in two databases

The Scatter plot illustrates the variations in specificity over time for two models. The results are presented for different prediction start times from the eICU-CRD and MIMIC-IV

Sensitive analysis

**Table S8.** Baseline characteristics of patients in four latent class in MIMIC-IV Sepsis-3 patients

|  | Class1 (N=1,816) | Class2 (N=17,894) | Class3 (N=562) | Class4 (N=1,069) | P |
| --- | --- | --- | --- | --- | --- |
| Basic Characteristics |  |  |  |  |  |
| Age (years), median (IQR) | 61.73 [48.48, 73.19] | 68.66 [57.76, 79.35] | 61.95 [50.38, 73.70] | 67.90 [57.09, 77.20] | <0.001 |
| Gender, n (%) |  |  |  |  | 0.003 |
| Female | 732 (40.3) | 7364 (41.2) | 251 (44.7) | 493 (46.1) |  |
| Male | 1084 (59.7) | 10530 (58.8) | 311 (55.3) | 576 (53.9) |  |
| Ethnicity, n (%) |  |  |  |  | <0.001 |
| Asian | 48 (2.6) | 518 (2.9) | 12 (2.1) | 27 (2.5) |  |
| Black/African American | 144 (7.9) | 1487 (8.3) | 30 (5.3) | 80 (7.5) |  |
| Caucasian | 1143 (62.9) | 12226 (68.3) | 356 (63.3) | 676 (63.2) |  |
| Hispanic/Latino | 80 (4.4) | 631 (3.5) | 18 (3.2) | 47 (4.4) |  |
| Native American | 4 (0.2) | 59 (0.3) | 1 (0.2) | 5 (0.5) |  |
| Other/Unknown | 397 (21.9) | 2973 (16.6) | 145 (25.8) | 234 (21.9) |  |
| Admission Unit type, n (%) |  |  |  |  | <0.001 |
| Medical Intensive Care Unit (MICU) | 358 (19.7) | 3831 (21.4) | 103 (18.3) | 300 (28.1) |  |
| Surgical Intensive Care Unit (SICU) | 992 (54.6) | 7370 (41.2) | 282 (50.2) | 462 (43.2) |  |
| Cardiac related Intensive Care Unit | 407 (22.4) | 6126 (34.2) | 167 (29.7) | 289 (27.0) |  |
| Neuro related Intensive Care Unit | 59 (3.2) | 567 (3.2) | 10 (1.8) | 18 (1.7) |  |
| BMI (kg/m^2^), median (IQR) | 27.97 [24.39, 32.75] | 28.34 [24.62, 32.99] | 28.03 [23.93, 32.96] | 27.49 [23.78, 32.73] | 0.006 |
| Weight (kg), median (IQR) | 81.00 [68.30, 96.60] | 80.05 [67.60, 95.50] | 80.00 [65.50, 97.70] | 78.00 [65.61, 95.00] | 0.106 |
| Height (cm), median (IQR) | 170.00 [163.00, 178.00] | 170.00 [163.00, 178.00] | 170.00 [163.00, 178.00] | 169.50 [160.00, 178.00] | 0.002 |
| Illness Severity |  |  |  |  |  |
| APS-Ⅲ, median (IQR) | 59.00 [44.00, 78.00] | 46.00 [34.00, 63.00] | 77.00 [54.00, 101.00] | 75.00 [55.00, 96.00] | <0.001 |
| SOFA, median (IQR) | 8.00 [5.00, 10.00] | 5.00 [4.00, 8.00] | 10.00 [7.00, 13.00] | 9.00 [6.00, 12.00] | <0.001 |
| Laboratory |  |  |  |  |  |
| Platelet Count(×109/L), median (IQR) | 120.00 [69.00, 175.00] | 160.00 [112.00, 223.00] | 156.00 [103.00, 223.50] | 199.00 [129.00, 298.00] | <0.001 |
| INR, median (IQR) | 1.20 [1.10, 1.40] | 1.20 [1.10, 1.40] | 1.20 [1.10, 1.40] | 1.30 [1.10, 1.60] | <0.001 |
| White Blood Cell(×109/L), median (IQR) | 9.10 [5.90, 12.50] | 9.60 [6.90, 13.00] | 10.50 [6.82, 14.78] | 11.70 [7.90, 16.50] | <0.001 |
| Hematocrit (%), median (IQR) | 27.70 [23.50, 32.70] | 29.30 [25.10, 33.80] | 29.15 [24.40, 35.48] | 28.20 [23.90, 33.90] | <0.001 |
| Calcium (mg/dL), median (IQR) | 7.70 [7.10, 8.20] | 8.00 [7.50, 8.50] | 7.50 [6.90, 8.10] | 7.80 [7.20, 8.50] | <0.001 |
| Bilirubin (mg/dL), median (IQR) | 0.80 [0.50, 1.50] | 0.70 [0.40, 1.40] | 0.60 [0.40, 1.20] | 0.70 [0.40, 1.80] | <0.001 |
| Creatinine (mg/dL), median (IQR) | 0.90 [0.70, 1.30] | 0.90 [0.70, 1.40] | 1.00 [0.70, 1.60] | 1.20 [0.80, 2.10] | <0.001 |
| BUN (mg/dL), median (IQR) | 17.00 [11.00, 27.00] | 18.00 [13.00, 30.00] | 19.00 [13.00, 30.00] | 26.00 [16.00, 44.00] | <0.001 |
| Blood Gas |  |  |  |  |  |
| PaO_2_ (mmHg), median (IQR) | 71.00 [46.00, 100.00] | 76.00 [45.00, 108.00] | 60.00 [40.00, 86.00] | 60.00 [39.00, 86.50] | <0.001 |
| PaCO_2_ (mmHg), median (IQR) | 34.00 [30.00, 38.00] | 35.00 [31.00, 40.00] | 32.00 [27.00, 36.00] | 33.00 [28.00, 39.00] | <0.001 |
| pH, median (IQR) | 7.30 [7.22, 7.37] | 7.32 [7.26, 7.38] | 7.21 [7.11, 7.30] | 7.28 [7.19, 7.36] | <0.001 |
| Base Excess (mEq/L), median (IQR) | -4.00 [-8.00, 0.00] | -2.00 [-6.00, 0.00] | -9.00 [-14.00, -4.00] | -6.00 [-10.00, -1.00] | <0.001 |
| Vital Signs |  |  |  |  |  |
| Heart Rate (/min), median (IQR) | 74.00 [64.00, 87.00] | 70.00 [60.00, 80.00] | 77.00 [64.25, 89.00] | 73.00 [62.00, 87.00] | <0.001 |
| Respiratory Rate (/min), median (IQR) | 13.00 [10.00, 15.00] | 12.00 [10.00, 15.00] | 13.00 [10.00, 15.38] | 13.00 [10.00, 16.00] | <0.001 |
| Temperature (℃), median (IQR) | 36.50 [36.11, 36.83] | 36.39 [35.94, 36.67] | 36.39 [35.41, 36.69] | 36.33 [35.61, 36.56] | <0.001 |
| SBP (mmHg), median (IQR) | 87.00 [78.00, 96.00] | 88.00 [80.00, 98.00] | 80.00 [69.00, 88.00] | 83.00 [75.00, 92.00] | <0.001 |
| DBP (mmHg), median (IQR) | 45.00 [40.00, 51.00] | 44.00 [39.00, 51.00] | 42.00 [37.00, 48.00] | 43.00 [36.00, 49.00] | <0.001 |
| Chronic Conditions |  |  |  |  |  |
| Charlson Comorbidity Score, median (IQR) | 5.00 [3.00, 7.00] | 6.00 [4.00, 8.00] | 5.00 [3.00, 7.00] | 6.00 [5.00, 8.00] | <0.001 |
| Chronic Pulmonary Disease, n (%) |  |  |  |  | <0.001 |
| No | 1415 (77.9) | 13144 (73.5) | 424 (75.4) | 754 (70.5) |  |
| Yes | 401 (22.1) | 4750 (26.5) | 138 (24.6) | 315 (29.5) |  |
| Diabetes, n (%) |  |  |  |  | <0.001 |
| No | 1695 (93.3) | 16154 (90.3) | 534 (95.0) | 978 (91.5) |  |
| Yes | 121 (6.7) | 1740 (9.7) | 28 (5.0) | 91 (8.5) |  |
| Chronic Renal Disease, n (%) |  |  |  |  | <0.001 |
| No | 1557 (85.7) | 13938 (77.9) | 477 (84.9) | 802 (75.0) |  |
| Yes | 259 (14.3) | 3956 (22.1) | 85 (15.1) | 267 (25.0) |  |
| Malignant Cancer, n (%) |  |  |  |  | <0.001 |
| No | 1593 (87.7) | 15452 (86.4) | 494 (87.9) | 868 (81.2) |  |
| Yes | 223 (12.3) | 2442 (13.6) | 68 (12.1) | 201 (18.8) |  |
| Intervention during ICU |  |  |  |  |  |
| Dialysis, n (%) |  |  |  |  | <0.001 |
| No | 1736 (95.6) | 17129 (95.7) | 493 (87.7) | 939 (87.8) |  |
| Yes | 80 (4.4) | 765 (4.3) | 69 (12.3) | 130 (12.2) |  |
| Invasive Mechanical Ventilation, n (%) |  |  |  |  | <0.001 |
| No | 490 (27.0) | 9673 (54.1) | 105 (18.7) | 250 (23.4) |  |
| Yes | 1326 (73.0) | 8221 (45.9) | 457 (81.3) | 819 (76.6) |  |
| Vasopressors, n (%) |  |  |  |  | <0.001 |
| No | 1565 (86.2) | 16618 (92.9) | 306 (54.4) | 651 (60.9) |  |
| Yes | 251 (13.8) | 1276 (7.1) | 256 (45.6) | 418 (39.1) |  |
| Antiplatelet Drugs, n (%) |  |  |  |  | <0.001 |
| No | 1566 (86.2) | 15617 (87.3) | 461 (82.0) | 821 (76.8) |  |
| Yes | 250 (13.8) | 2277 (12.7) | 101 (18.0) | 248 (23.2) |  |
| Antibiotics, n (%) |  |  |  |  | <0.001 |
| No | 345 (19.0) | 9014 (50.4) | 81 (14.4) | 131 (12.3) |  |
| Yes | 1471 (81.0) | 8880 (49.6) | 481 (85.6) | 938 (87.7) |  |
| Glucocorticoids, n (%) |  |  |  |  | <0.001 |
| No | 1709 (94.1) | 17023 (95.1) | 537 (95.6) | 973 (91.0) |  |
| Yes | 107 (5.9) | 871 (4.9) | 25 (4.4) | 96 (9.0) |  |
| Blood Transfusion, n (%) |  |  |  |  | <0.001 |
| No | 1117 (61.5) | 14997 (83.8) | 258 (45.9) | 492 (46.0) |  |
| Yes | 699 (38.5) | 2897 (16.2) | 304 (54.1) | 577 (54.0) |  |
| Platelet Transfusion, n (%) |  |  |  |  | <0.001 |
| No | 1642 (90.4) | 17226 (96.3) | 455 (81.0) | 856 (80.1) |  |
| Yes | 174 (9.6) | 668 (3.7) | 107 (19.0) | 213 (19.9) |  |
| Clinical Outcomes |  |  |  |  |  |
| Thrombocytopenia time^1^ | 0.36 (0.57) | 0.53 (1.23) | 1.08 (1.04) | 2.18 (2.59) | <0.001 |
| Severe Thrombocytopenia time^2^ | 0.36 (0.41) | 2.40 (4.29) | 2.80 (1.51) | 5.97 (3.17) | <0.001 |
| ICU length of stay (days), median (IQR) | 7.39 [4.53, 13.00] | 2.62 [1.73, 4.55] | 9.52 [3.86, 14.81] | 6.82 [4.41, 11.58] | <0.001 |
| Hospital length of stay (days), median (IQR) | 16.56 [10.86, 25.68] | 7.96 [5.16, 13.42] | 16.26 [7.99, 23.99] | 12.45 [7.30, 21.27] | <0.001 |
| Clinical outcome, n (%) |  |  |  |  | <0.001 |
| Discharged alive or censored at 28-day | 1697 (93.4) | 15849 (88.6) | 412 (73.3) | 601 (56.2) |  |
| Non-survivors | 119 (6.6) | 2045 (11.4) | 150 (26.7) | 468 (43.8) |  |

1: Thrombocytopenia time is defined as the time at which a patient's platelet measurement falls below 150×10^9 /L for the first time; 2: Severe Thrombocytopenia time is defined as the time at which a patient's platelet measurement falls below 20×10^9 /L for the first time.

**Figure S10.** Trajectory plots and KM survival curves of patients with four dynamic platelet count trajectory patterns.

Trajectory plots of platelet count changes within the first 28 days after ICU admission in the MIMIC-IV based on Sepsis-3, along with their corresponding survival curves.

**Table S9.** Time-dependent HR for four classes in the sensitivity analysis set

|  |  | HR (95% CI) | |
| --- | --- | --- | --- |
|  | Time Points (Day) | (0,3] | (3,28] |
| MIMIC-IV Sepsis-3 | Class2 (slight-increasing) | 4.66 (2.69, 8.07) | 2.92 (2.40, 3.56) |
|  | Class3 (decreasing-increasing-medium) | 16.91 (9.32, 30.70) | 2.90 (2.18, 3.85) |
|  | Class4 (decreasing-low) | 9.31 (5.15, 16.83) | 8.56 (6.91, 10.61) |

**Figure S11.** Time-dependent AUC for two models at different landmark in MIMIC-IV Sepsis-3 patients


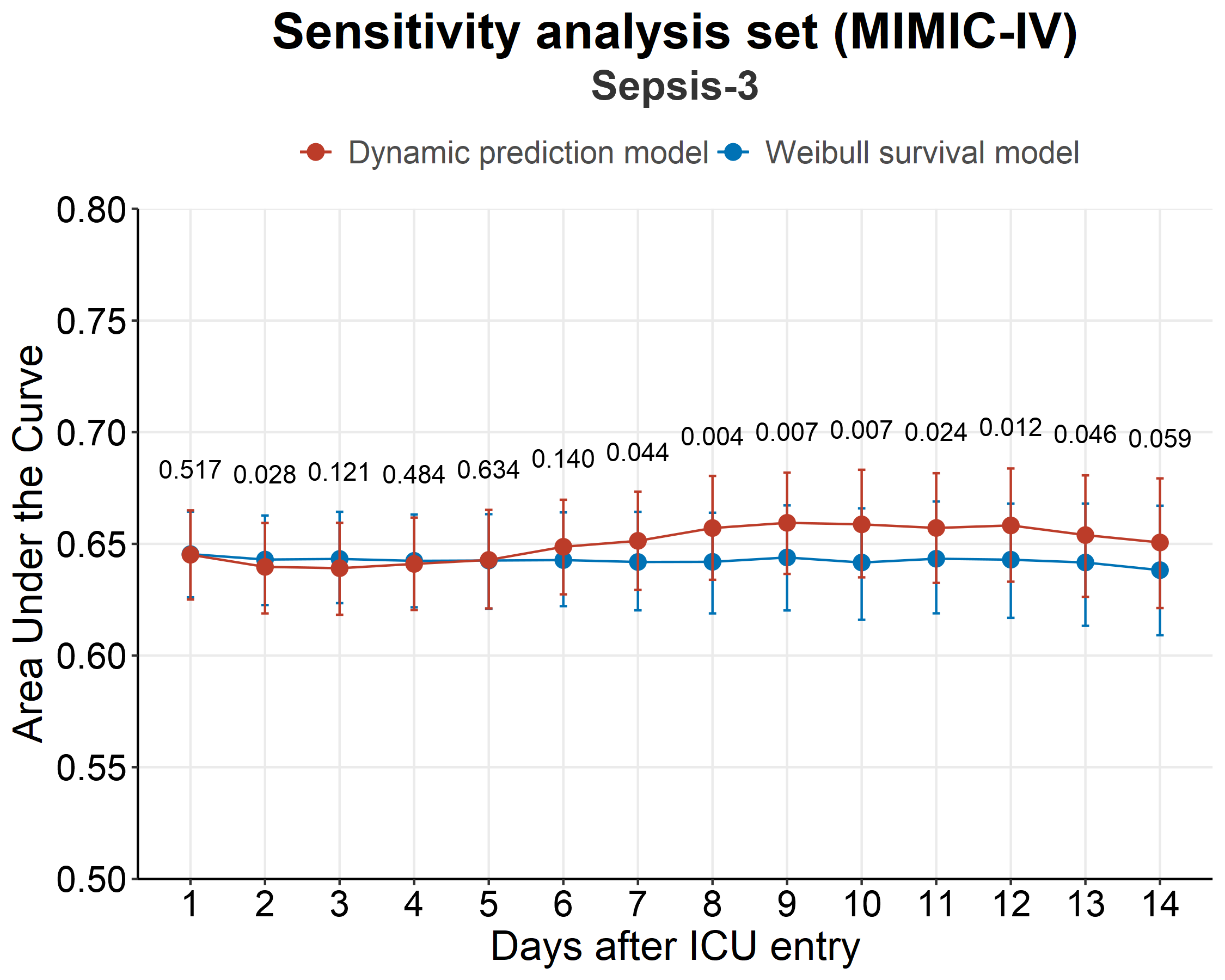


The numbers in the plot represent the p-values of the time-dependent AUC differences between two models, calculated through permutation tests by shuffling 2000 times at each landmark time.

**Figure S12.** C-index for two models at different landmark time in MIMIC-IV Sepsis-3 patients


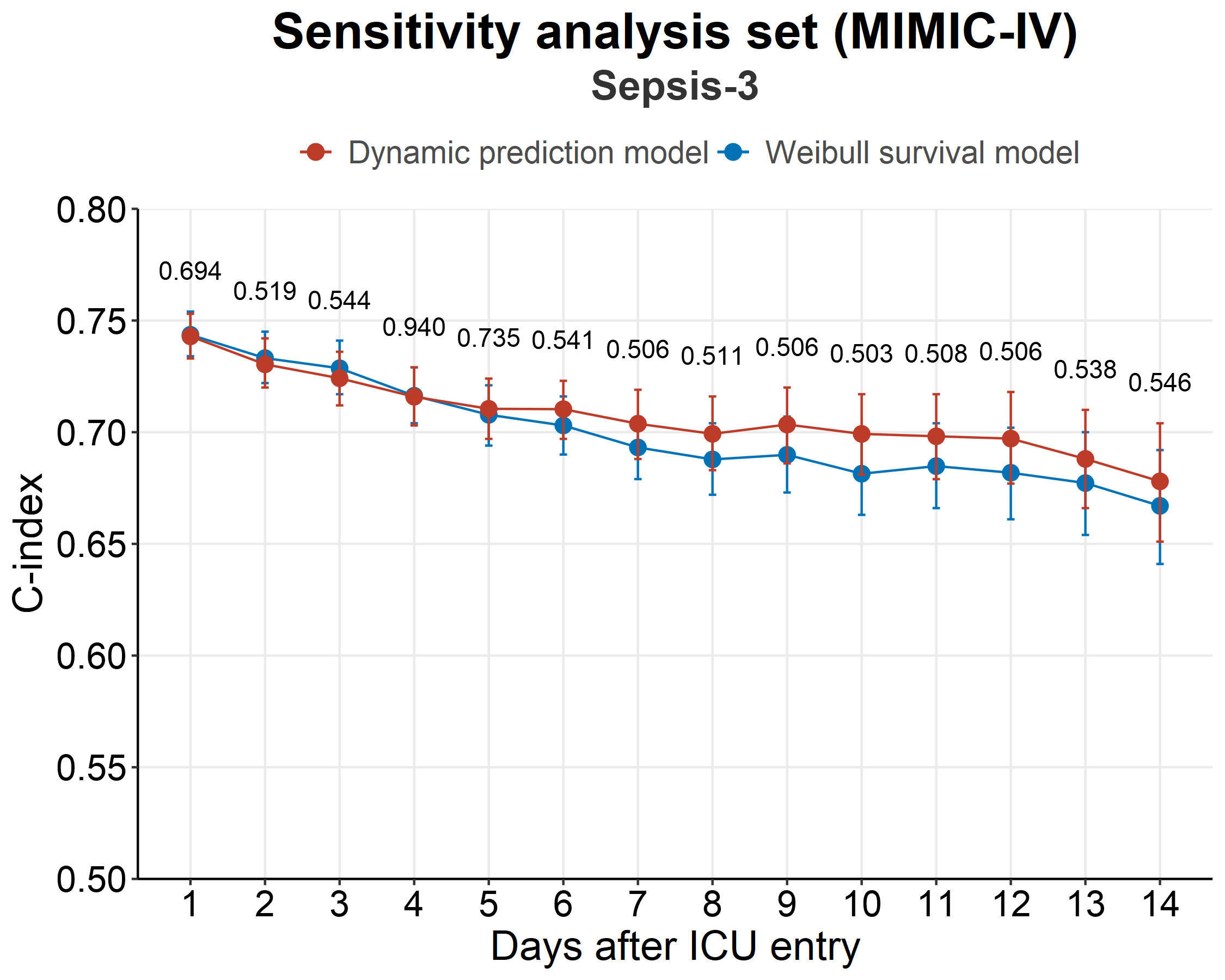


The numbers in the plot represent the p-values of the C-index differences between two models, calculated through permutation tests by shuffling 2000 times at each landmark time.

References

1. Proust-Lima C, Joly P, Dartigues J-F, Jacqmin-Gadda H. Joint modelling of multivariate longitudinal outcomes and a time-to-event: A nonlinear latent class approach. *Computational Statistics & Data Analysis* 2009, 53(4):1142-1154. doi: <https://doi.org/10.1016/j.csda.2008.10.017>.

2. Lin H, Turnbull BW, McCulloch CE, Slate EH. Latent Class Models for Joint Analysis of Longitudinal Biomarker and Event Process Data. *Journal of the American Statistical Association* 2002, 97(457):53-65. doi: 10.1198/016214502753479220.

3. Proust-Lima C, Taylor JM. Development and validation of a dynamic prognostic tool for prostate cancer recurrence using repeated measures of posttreatment PSA: a joint modeling approach. *Biostatistics* 2009, 10(3):535-549. doi: 10.1093/biostatistics/kxp009.

4. Celeux G, Soromenho G. An entropy criterion for assessing the number of clusters in a mixture model. *Journal of Classification* 1996, 13(2):195-212. doi: 10.1007/BF01246098.

5. Ram N, Grimm KJ. Methods and Measures: Growth mixture modeling: A method for identifying differences in longitudinal change among unobserved groups. *International Journal of Behavioral Development* 2009, 33(6):565-576. doi: 10.1177/0165025409343765.

6. Ramaswamy V, DeSarbo WS, Reibstein DJ, Robinson WT. An Empirical Pooling Approach for Estimating Marketing Mix Elasticities with PIMS Data. *Marketing Science* 1993, 12:103-124. doi.

7. Wong GYC, Osborne MP, Diao Q, Yu Q. Piecewise Cox models with right-censored data. *Communications in Statistics - Simulation and Computation* 2017, 46(10):7894-7908. doi: 10.1080/03610918.2016.1255968.
